# Integrated pan-cancer analysis reveals the immunological and prognostic potential of RBFOX2 in human tumors

**DOI:** 10.3389/fphar.2024.1302134

**Published:** 2024-05-31

**Authors:** Fengxian Huang, Long Jin, Xinyue Zhang, Min Wang, Congya Zhou

**Affiliations:** ^1^ Department of Radiation Oncology, Shaanxi Provincial People’s Hospital, Xi’an, China; ^2^ Department of Science and Education, Xi’an Children’s Hospital Affiliated of Xi’an Jiaotong University, Xi’an, China

**Keywords:** pan-cancer, RBFOX2, tumor immune microenvironment, immunotherapy, methylation

## Abstract

**Background:**

The role of RNA-binding fox one homolog 2 (RBFOX2) in the progression of multiple tumors is increasingly supported by evidence. However, the unclearness pertaining to the expression of RBFOX2, its prognostic potential, and its correlation with the tumor microenvironment (TME) in pan-cancer persists. This study aims to comprehensively investigate the immunological prognostic value of RBFOX2.

**Methods:**

The Cancer Genome Atlas Gene Expression Omnibus Genotype-Tissue Expression (GTEx), TIMER2.0, Kaplan-Meier (K–M) Plotter, University of Alabama at Birmingham Cancer data analysis Portal (UALCAN), cbioportal, and Gene Expression Profiling Interactive Analysis 2 (GEPIA2) were utilized for a systematic analysis of RBFOX2. This analysis included studying its expression, prognostic value, DNA methylation, enrichment analysis, immune infiltration cells, and immune-related genes. Additionally, qRT-PCR, CCK-8, colony formation, transwell assays, and immunohistochemistry were employed to analyze the expression and biological function of RBFOX2 in liver cancer.

**Results:**

Variations in RBFOX2 expression have been observed across diverse tumors and have been identified as indicators of unfavorable prognosis. It is closely linked to immune infiltration cells, immune checkpoints, chemokines, and chemokine receptors in the TME. Higher levels of RBFOX2 have been significantly associated with low response and poor prognosis in patients with non-small cell lung cancer (NSCLC) and melanoma who receive immunotherapy. Furthermore, the DNA methylation of RBFOX2 varies across different types of cancer and has shown better prognosis in patients with BLCA, BRCA, CESC, COAD, DLBC, HNSC, LAML, LGG, LUAD, PAAD, SKCM and THYM. Interestingly, RBFOX2 expression was found to be lower in hepatocellular carcinoma (HCC) patients’ tumor tissues compared to their paired adjacent tissues. *In vitro* studies have shown that knockdown of RBFOX2 significantly promotes the growth and metastasis of liver cancer cells.

**Conclusion:**

This study investigates the correlation between DNA methylation, prognostic value, and immune cell infiltration with the expression of RBFOX2 in pan-cancer and indicates its potential role to inhibit metastasis of liver cancer.

## 1 Introduction

Cancerous growths are a prevalent catalyst of mortality on a global scale (Sung et al., 2021). Despite endeavors to enhance treatment and prognosis, the overall survival (OS) for patients remains unsatisfactory (Ferlay et al., 2021). The present options for cancer therapy encompass surgical intervention, chemotherapy, radiotherapy, targeted therapy, and immunotherapy. However, the efficacy of immunotherapy is restrained by the high heterogeneity of the tumor immune microenvironment (TME) (Zhang and Zhang, 2020; Li et al., 2023; Sun et al., 2023). Therefore, identifying novel prognostic and immune-related biomarkers is crucial for enhancing the success rate of cancer therapy.

RNA-binding fox one homolog 2 (RBFOX2), a well-known member of RNA-binding fox (RBFOX) family, binds to the GCAUG element to regulate alternative splicing (Jin et al., 2003; Singh et al., 2014; Kim et al., 2021). RBFOX2 is involved in proliferation, epithelial–mesenchymal transition (EMT), differentiation and tumor development (Arya et al., 2014). Its association with the EMT process in ovarian and breast cancer is of utmost importance in facilitating cancer metastasis (Shapiro et al., 2011; Zhou et al., 2021). There have been reports on the enhancement of laryngeal cancer cells’ metastasis through the upregulation of RBFOX2, achieved by skipping exon 11a of MENA (Lu et al., 2023). Furthermore, RBFOX2 controls splicing alterations that induce a transition from epithelial to mesenchymal states, thus contributing to the manifestation of an invasive phenotype (Braeutigam et al., 2014). Furthermore, RBFOX2 affects the expression of Hippo-YAP pathway by regulating the splicing of TEAD1 exon 6, thereby increasing the oncogenic properties of cancer (Choi et al., 2022). RBFOX2 has been reported to alter the splicing of GOLIM4 in nasopharyngeal carcinoma (Luo et al., 2021). Recent studies have demonstrated that RBFOX2 regulates the alternative splicing event of MPRIP, showing tumor suppressor potential for metastatic pancreatic cancer (Jbara et al., 2023). Additionally, RBFOX2 promotes N6-methyladenosine (m6 A) methylation by recruiting RBM15 to chromatin-associated RNAs. Downregulation of RBFOX2 inhibits growth of acute myeloid leukemia cells and promotes the differentiation (Dou et al., 2023). While there have been prior investigations emphasizing the significance of RBFOX2 in the context of cancer, a holistic evaluation encompassing multiple cancer types is still lacking. Accordingly, the objective of this study is to conduct an analysis encompassing the expression patterns, prognostic implications, and correlation with the immune system in a diverse range of 33 cancer types.

In this research, we investigated the expression and prognosis of RBFOX2, as well as its correlation with the infiltration of immune cells, immune checkpoint genes, and other markers related to the immune system, including chemokines,chemokine receptor, and genes coding for major histocompatibility complex (MHC). Additionally, we analyzed the methylation level of RBFOX2 and its association with patient prognosis, shedding light on the potential mechanism underlying the differential expression of RBFOX2 in various tumors. Notably, we observed lower levels of RBFOX2 in tumor tissues of hepatocellular carcinoma (HCC) patients compared to paired adjacent tissues. In addition, the results indicated a considerable enhancement in both the proliferation and metastasis of liver cancer cells following RBFOX2 knockdown. Our discoveries propose the potential utilization of RBFOX2 as a predictive marker, emphasizing its pivotal involvement in the tumor microenvironment across various malignancies, thereby underscoring its potential influence on cancer immunotherapy.

## 2 Materials and methods

### 2.1 Data collection

The UCSC Xena database (https://xenabrowser.net/datapages/) provided access to the RNA expression, DNA methylation, and relevant clinical data (including age, gender, and tumor stage) of pan-cancer patients obtained from The Cancer Genome Atlas (TCGA). Additionally, corresponding normal tissues from Genotype-Tissue Expression (GTEx) were downloaded for comparison. RNA sequencing data for RBFOX2 in various cancer cell lines were retrieved from the Cancer Cell Line Encyclopedia (CCLE) project through the DepMap portal accessible at https://depmap.org/portal/download/all/. The RNA expression levels were standardized as transcripts per million (TPM) and log normalized, while DNA methylation levels were assessed using average beta values for the promoter regions. To assess RBFOX2 expression in pan-cancer and normal tissues, the UALCAN database at http://ualcan.path.uab.edu/analysis-prot.html was utilized. Additionally, the GSE135222 dataset was obtained from the Gene Expression Omnibus (GEO) database, and the IMvigor210 dataset was sourced from the R package “IMvigor210CoreBiologies.” The PMID30753825 and PMID32472114 datasets were downloaded from the published work (Gide et al., 2019; Braun et al., 2020).

### 2.2 Survival analysis

In the investigation of the prognostic impact of RBFOX2 in pan-cancer, four categories of patients’ survival data were examined, specifically OS, disease specific survival (DSS), disease free interval (DFI) and progression free interval (PFI). Besides, the optimal cutoff point was determined using the surv_cutpoint function within the R package survminer, and graphically showcased using Kaplan-Meier (K–M) curves. Statistically significant was deemed if the calculated *p*-value was less than 0.05.

### 2.3 Tumor microenvironment estimation

We estimated the tumor purity, stromal score, immune score, and ESTIMATE score of patients in pan-cancer cohorts by employing the R package. The pre-calculated immune cell infiltration of each patient were extracted from several well-known resources including TIMER2 (http://timer.cistrome.org/), ImmuCellAI (http://bioinfo.life.hust.edu.cn/ImmuCellAI), and a previous published work (Thorsson et al., 2018). Additionally, the proportion of 22 common immune cell types were predicted by traditional markers with the help of R package CIBERSORT.

### 2.4 Gene set enrichment analysis

Metabolic pathways were retrieved from KEGG and the R package GSVA was applied to calculate metabolic activities using single sample gene set enrichment analysis (ssGSEA) mode at pan-cancer level.

### 2.5 Correlation analysis

The correlation between RBFOX2 expression and all above tumor microenvironment indicators, as well as the expression of immune-activating genes, immunosuppressive genes, chemokine receptor, chemokine, MHC families, and metabolic pathways were computed by Pearson correlation analysis. Statistically significant was deemed if the calculated *p*-value was less than 0.05.

### 2.6 RBFOX2-related genes analysis

The Protein-Protein Interactions (PPI) between RBFOX2 and other genes were searched from Genemina (http://genemania.org/) and STRING (https://cn.string-db.org/) with default parameters. These proteins predicted to be interact with RBFOX2 were defined as RBFOX2-related genes. Then PPI networks were exported and the Cytoscape software was used to visualize this result. The R package clusterProfiler was used to carry out gene annotations for RBFOX2-related genes in terms of Gene Ontology (GO) and Kyoto Encyclopedia of Genes and Genomes (KEGG) pathway.

### 2.7 Immunohistochemical (IHC) and staining results

The microarray slides, containing HCC tumor tissues and paired adjacent tissues, were acquired from WEIAO Biotech, Ltd. (Shanghai, China). In order to assess the protein expression, an IHC experiment was conducted employing the RBFOX2 primary antibody (Proteintech, Cat#12498-1-AP, diluted at 1:200) following a standardized protocol. The intensity of RBFOX2 staining was divided into 0 (absence), 1 (low), 2 (moderate), and 3 (high). Grades 0 and 1 were considered as low expression, whereas grades 2 and 3 were classified as high expression. To determine the RBFOX2 expression score, the following formula was employed: percentage of low staining multiplied by 1, plus percentage of moderate staining multiplied by 2, plus percentage of high staining multiplied by 3 (Kim et al., 2016).

### 2.8 Cell culture and gene knockdown

The HepG2, Huh7, and 293T cells were acquired from the National Collection of Authenticated Cell Cultures (Shanghai, China). These cells were cultivated at 37°C with 5% CO2 in DMEM medium enriched with 10% serum, 100 U/mL penicillin (purchased from Beyotime, China), and 100 mg/mL streptomycin (obtained from Beyotime, China).

Gene knockdown assays were performed according to Hu et al. (2019). The sequence for shRBFOX2#1 was GGG​TTC​GTA​ACT​TTC​GAG​AA, the sequence for shRBFOX2#2 was TTG​GCG​CTG​TGG​CGA​GTT​TAT.

### 2.9 Western blotting

After whole cell lysates was put on ice for 30 min incubated with Radio-Immune Precipitation Assay (RIPA) lysis buffer. Fractionation of the protein lysates was carried out using 10% SDS-PAGE. Following fractionation, the proteins were subsequently transferred onto PVDF membranes. For detection, RBFOX2 primary antibodies (diluted 1:1,000) were employed and allowed to incubate overnight at 4°C, following a 1-h blocking step using blocking buffer at room temperature. GAPDH was used as control. Subsequent washing of the PVDF membrane was performed three times, followed by a 2-h incubation with secondary antibodies at room temperature. Further washing of the PVDF membranes was performed three additional times before visualization was achieved using enhanced chemiluminescence.

### 2.10 CCK-8 assay

HepG2 and Huh7 cells treated with control or shRBFOX2 virus were seeded into 96-well plates (2000 cells per well). Ten microliters of CCK8 reagent (TargetMol, Cat#C0005) were added to and incubated for 3 h. The absorbance of 450 nm was measured using a multifunction microplate reader (BioTeK) at 0, 24 and 48 h.

### 2.11 Colony formation assay

After preparing single cell suspensions, 12-well plates were used to seed a total of 4,000 control or shRBFOX2 cells per well. Following a 14-day incubation period, the culture medium was eliminated, and PBS was used to wash the cells. To fix the cells, a 20-min treatment with formaldehyde (Sigma-Aldrich) was applied, followed by another round of PBS washing. Subsequently, the cells were subjected to a 30-min staining with crystal violet (Sigma-Aldrich). After being washed with deionized water, the cells were scanned.

### 2.12 Transwell assay

Transwell chambers were used to performed migration and invasion experiments. After transfected with control or shRBFOX2 virus and selected by puromycin for 48 h, total of 200000 HepG2 and Huh7 cells were seeded in upper chamber. To conduct the plagiarism check, slight modifications will be made to the original text. The DMEM medium, which contained 0.05% FBS, was positioned in the upper chamber of the transwell plate (24-well, 8 μm, Corning, Life Sciences), whereas the lower chamber housed the DMEM medium with a concentration of 10% FBS. When performing the invasion assay, Matrigel (BD Biosciences, United States) was incorporated into the upper chambers; while the migration assay was conducted without the use of Matrigel. Subsequent to a 24-h duration, the non-migrating cells were delicately eliminated, leaving only the invasive and migrated cells. These cells were then fixed by employing 4% paraformaldehyde, stained with 1% crystal violet, and subsequently subjected to scanning for analysis.

### 2.13 Statistical analysis

Statistical analyses were conducted using R software version 4.0.2 (https://www.r-project.org/). In order to assess variations in expression between two groups, the Wilcoxon rank sum test was employed. The survival distributions of the two groups were compared using the log rank test. Data analysis was performed using GraphPad Prism 6. A two-tailed Student’s *t*-test was utilized to evaluate differences between the two groups. *p* < 0.05 was considered significant.

## 3 Results

### 3.1 RBFOX2 expression analysis in pan-cancer

**FIGURE 1:**
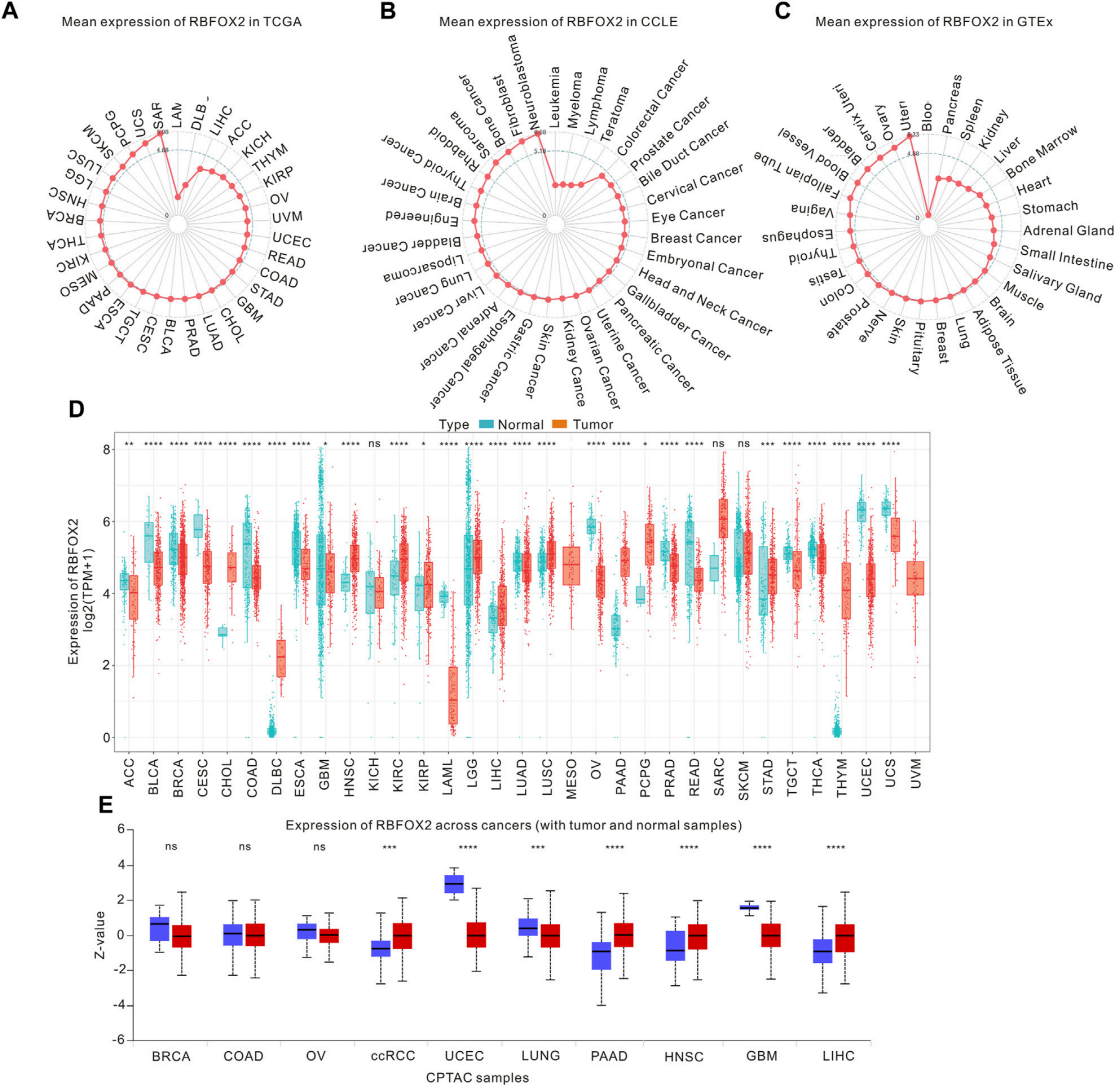
RBFOX2 expression in pan-cancer. **(A)** The mRNA levels of RBFOX2 in tumor tissues was analyzed using data from the TCGA database. **(B)** The mRNA levels of RBFOX2 in tumor cells was examined using data from the CCLE database. **(C)** The mRNA levels of RBFOX2 in normal tissues from the GTEx database. The dot’s position on the graph indicates the average RBFOX2 expression level. **(D)** Pan-cancer mRNA levels of RBFOX2 between tumor tissues from TCGA database with normal tissues from TCGA and GTEx databases. **(E)** The protein expression of RBFOX2 in tumor and normal tissues from CPTAC database. ccRCC, clear cell renal cell carcinoma; *, *p* < 0.05; **, *p* < 0.01; ***, *p* < 0.001, ****, *p* < 0.0001; ns, not significant.

In this investigation, we examined the expression of RBFOX2 across various cancers using the TCGA and CCLE database. Figure 1A illustrated the variations in RBFOX2 levels, with the highest being observed in SARC and the lowest in LAML. Moreover, we observed that RBFOX2 expression was elevated in neuroblastoma and reduced in leukemia cells (Figure 1B). In terms of normal tissues, RBFOX2 exhibited the highest expression in the uterus, while the lowest expression was detected in blood samples (Figure 1C). It is found that RBFOX2 was increased in CHOL, DLBC, HNSC, KIRC, KIPP, LGG, LIHC, LUSC, PAAD, PCPG, STAD, and THYM, while RBFOX2 was decreased in ACC, BLCA, BRCA, CESC, COAD, ESCA, GBM, LAML, LUAD, OV, PRAD, PEAD, TGCT, THCA, UCEC, and UCS (Figure 1D). Moreover, we also detected the RBFOX2 expression in various types of cancer and normal tissues. Our findings, as depicted in Figure 1E, revealed higher expression of RBFOX2 protein in clear cell renal cell carcinoma (ccRCC), PAAD, HNSC, and LIHC. Conversely, the RBFOX2 protein was lower in UCEC, LUNG and GBM.

**FIGURE 2:**
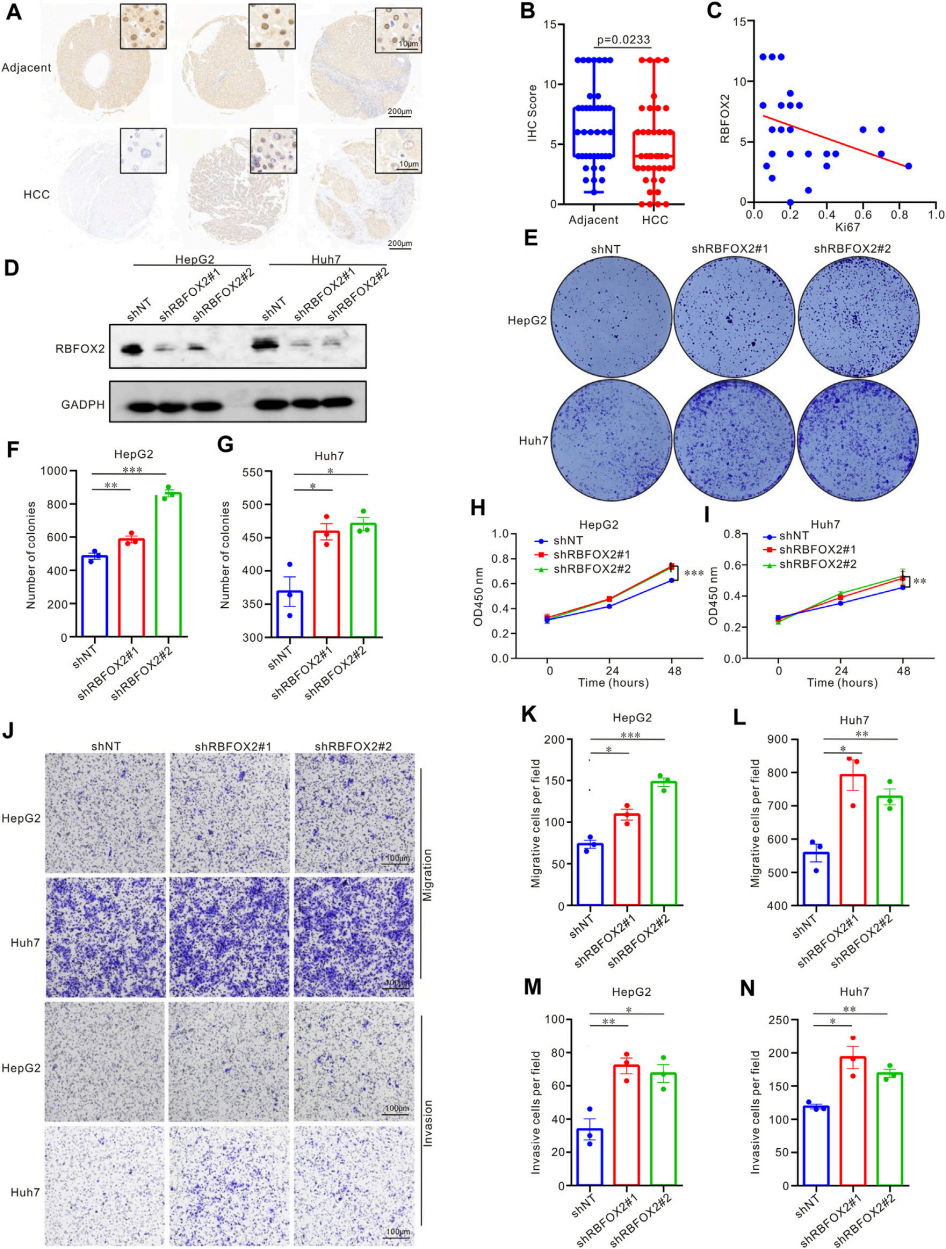
RBFOX2 expression and biological effect in HCC patients and in liver cancer cell lines. **(A)** RBFOX2 expression in HCC tumor tissues and paired adjacent tissues with IHC staining. **(B)** Microarray analysis of RBFOX2 levels in HCC tumor tissues compared to paired adjacent tissues (*n* = 40). **(C)** The correlation between RBFOX2 with Ki67 in HCC tumor tissues. **(D)** The protein of RBFOX2 levels in the HepG2 and Huh7 cells transfected with the sh-RBFOX2. Two short hairpin RNA targeting RBFOX2 was transfected to construct the knockdown of RBFOX2. **(E–G)** RBFOX2 knockdown significantly increased HepG2 and Huh7 cells colony formation ability. RBFOX2 knockdown significantly increased HepG2 **(H)** and Huh7 **(I)** cells proliferation. **(J–L)** RBFOX2 knockdown promoted the migration ability of HepG2 and Huh7 cells from Transwell assay. **(J,M,N)** RBFOX2 knockdown promoted the invasion ability of HepG2 and Huh7 cells from Transwell assay. HCC, hepatocellular carcinoma; IHC, immunohistochemistry; shNT, control group; *, *p* < 0.05; **, *p* < 0.01; ***, *p* < 0.001.

To delve deeper into the expression and biological effects of RBFOX2, we evaluated its expression in HCC patients’ tumor tissues and paired adjacent tissues (Figures 2A,B). Our experimental findings showcased decreased RBFOX2 expression in HCC tissues in contrast to their corresponding adjacent tissues. Moreover, we identified a notable inverse association between RBFOX2 expression and Ki67 expression (Figure 2C). In Table 1, we have summarized our exploration of the association between RBFOX2 expression and various clinical characteristics. Notably, our findings unveiled an inverse relationship between RBFOX2 expression and both the T stage and tumor size (*p* = 0.024 for both). In addition, we generated RBFOX2-knockdown liver cancer cell lines (Figure 2D). We assessed the impact of RBFOX2 knockdown on colony formation and cell proliferation in HepG2 and Huh7 cells (Figures 2E–I). Notably, knockdown of RBFOX2 led to an increase in colony numbers and enhanced proliferation of liver cancer cells. In addition, we examined the influence of RBFOX2 on the control of migration and invasion capacities of liver cancer cells through the utilization of Transwell experiments (Figure 2J–N). Our findings demonstrated that RBFOX2 knockdown led to heightened rates of migration and invasion in HepG2 and Huh7 cells. All in all, our discoveries suggested that RBFOX2 assumes a function in impeding the progression and metastasis of liver cancer cells *in vitro*.

**TABLE 1:** Association of RBFOX2 expression and clinicopathologic parameters.

Parameters		n	Low expression	High expression	X^2^	*p*-Value
Number of patients		40	16	24		
Age	≤60	18	9	9	1.364	0.243
	>60	22	7	15		
Sex	Male	30	12	18	0.000	1.000
	Female	10	4	6		
T stage	1–2	20	4	16	5.104	0.024^a^
	3–4	20	12	8		
Size	≤5 cm	20	4	16	5.104	0.024^a^
	>5 cm	20	12	8		
Metastasis	Yes	5	4	1	2.143	0.143
	No	35	12	23		
Grade	2–3	32	12	20	0.059	0.809
	4	8	4	4		
Preoperative AFP value	Normal	29	13	16	0.423	0.515
	Abnormal	11	3	8		

^a^ *p* < 0.05 was considered statistically significant.

### 3.2 Elevated RBFOX2 expression was correlated with shorter PFI in ACC, BLCA, BRCA, CESC, HNSC, LUAD, and UVM

**FIGURE 3:**
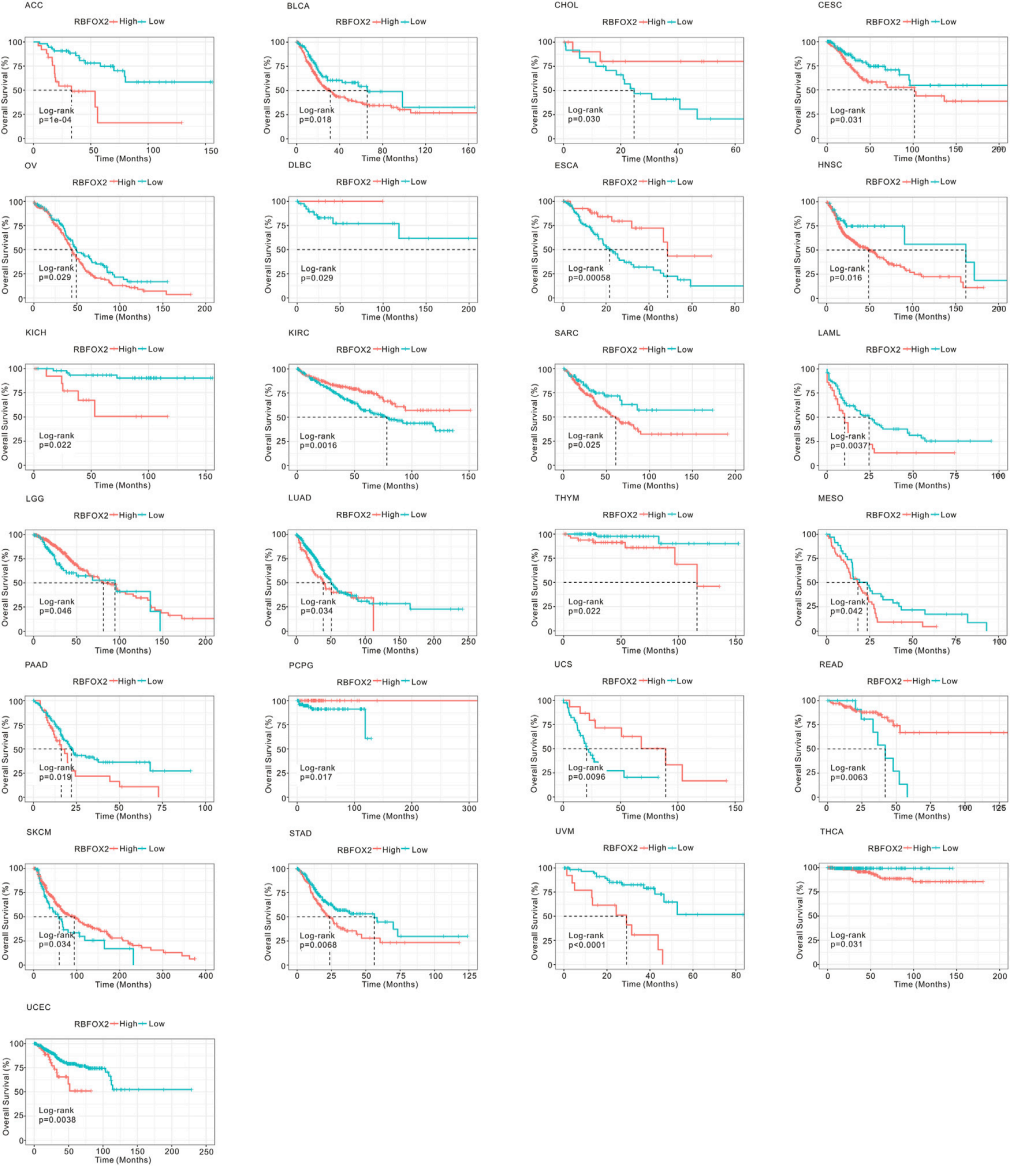
Association of RBFOX2 expression with OS in pan-cancer. Kaplan-Meier analysis of the relationship between the OS of cancer patients and RBFOX2 expression from TCGA database. The optimal cut-off value of expression of RBFOX2 was determined by surv_cutpoint function. OS, overall survival.

In order to assess the prognostic effect of RBFOX2, we utilized TCGA datasets to analyze OS, DSS, DFS and PFI in pan-cancer. As shown in Figure 3 and Supplementary Figure S1, our findings indicated that increased levels of RBFOX2 corresponded to unfavorable OS outcomes in multiple cancer types including ACC, BLCA, CESC, HNSC, KICH, LAMI, LUAD, MESO, OV, PAAD, SARC, STAD, THCA, THYM, UCEC, and UVM. Conversely, higher levels of RBFOX2 were linked to better OS in CHOL, DLBC, ESCA, KIRC, LGG, PCPG, READ, SKCM, and UCS. Additionally, we assessed DSS, DFS, and PFI in relation to RBFOX2 expression across various cancer types. Our results showed that patients with high RBFOX2 levels generally had poor DSS in ACC, BLCA, CHOL, COAD, HNSC, KICH, LUAD, MESO, OV, PAAD, PCPG, SKLM, STAD, UCEC, and UVM (Supplementary Figure S2). Furthermore, increased RBFOX2 levels were associated with shorter DFS in ACC, BLCA, COAD, KIRP, OV, PAAD, PCPG, and UCEC patients (Supplementary Figure S3). Finally, our analysis of PFI data demonstrated that elevated RBFOX2 levels were correlated with shorter PFI in ACC, BLCA, BRCA, CESC, HNSC, LUAD, and UVM patients (Supplementary Figure S4).

All in all, RBFOX2 was differently expressed in several of tumors, and its correlation with OS of different tumors was different (Supplementary Table S1). We summarized the expression of RBFOX2 and its correlation with OS in pan-cancer and found that higher RBFOX2 in HNSC, PAAD, STAD and THYM was consistent with its poor indicator for OS.. And for ESCA, READ and UCS, lower RBFOX2 was consistent with better indicator for OS..

### 3.3 Association of RBFOX2 expression and tumor immune microenvironment

**FIGURE 4:**
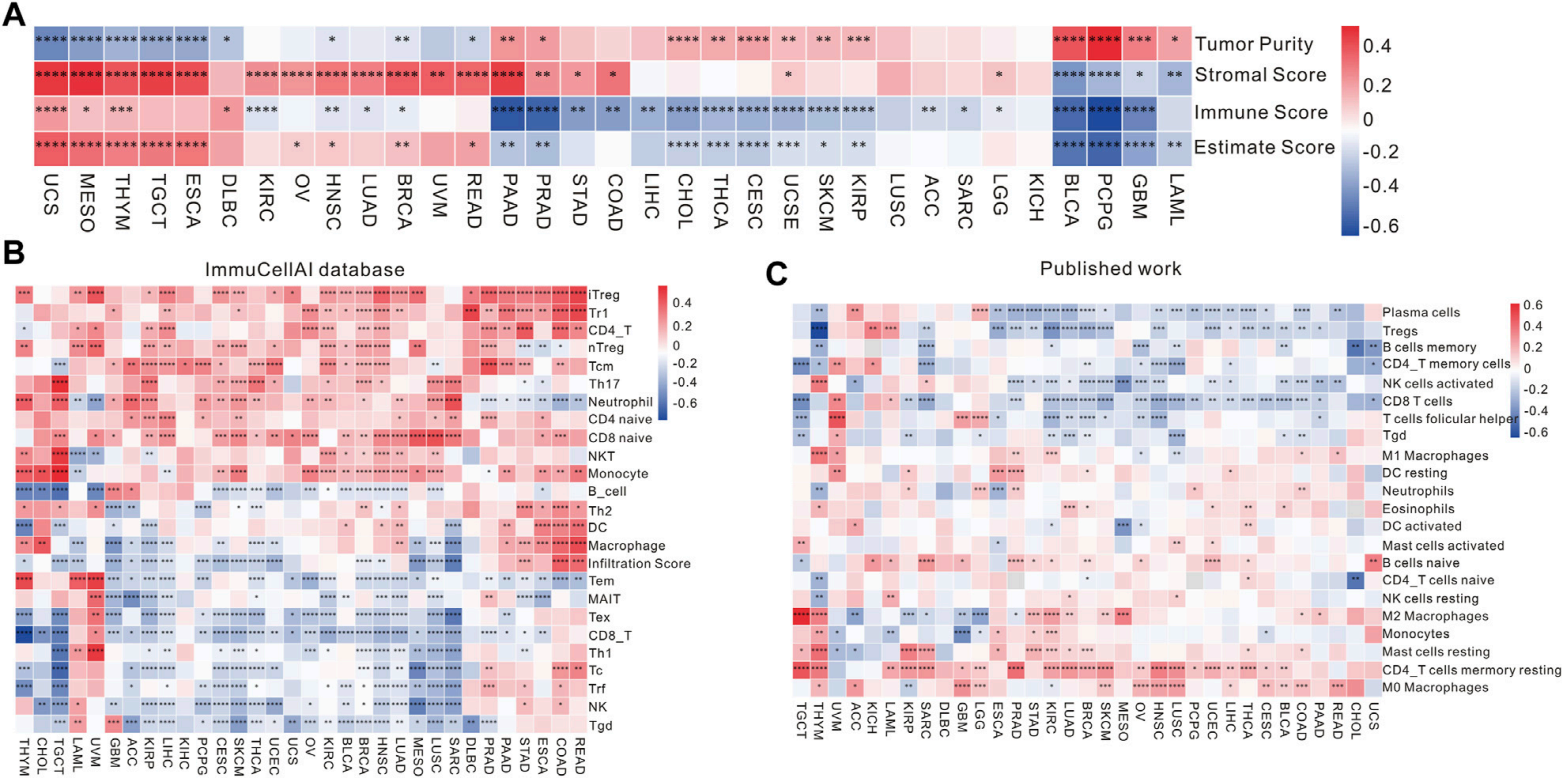
Association of RBFOX2 expression with tumor microenvironment and immune cell infiltration. **(A)** A heatmap of the association between RBFOX2 expression and Tumor Purity, Stromal Score, Immune Score and Estimate Score. **(B)** Examining the association between RBFOX2 expression with immune cell infiltration using the ImmuCellAI database. **(C)** Examining the association between RBFOX2 expression with immune cell infiltration from published work (PMID29628290). *, *p* < 0.05; **, *p* < 0.01; ***, *p* < 0.001, ****, *p* < 0.0001.

In order to examine the impact of RBFOX2 on the immune response of tumors, we conducted an analysis investigating the association between RBFOX2 and immune score, stromal score, and tumor purity score. As shown in Figure 4 and Supplementary Figure S5, our findings indicate that there exists a positive correlation between RBFOX2 and tumor purity score, while a negative correlation was observed between RBFOX2 and immune score as well as stromal score in LAML, GBM, PCPG, and BLCA. Conversely, UCS, MESO, and THYM exhibited a negative correlation between RBFOX2 and tumor purity score, while a positive correlation was observed between RBFOX2 and immune score as well as stromal score. Additionally, in PAAD, PRAD, and THYM, RBFOX2 displayed a positive correlation with tumor purity score and stromal score, while having a negative correlation with immune score. In the cases of HNSC and BRCA, RBFOX2 showed a negative correlation with both tumor purity score and immune score, but a positive correlation with stromal score. Notably, RBFOX2 exhibited significant correlations with tumor purity score and estimate score across various pan-cancer types. Overall, these results suggested that RBFOX2 may influence the development of pan-cancer by actively participating in and interacting with the TME.

In order to delve deeper into the relationship between RBFOX2 expression and immune cells in TME, we performed correlation analysis using various databases. In the ImmuCellAI database, within the realm of pan-cancer, RBFOX2 exhibited a negative correlation with the gamma delta T cells, NK cells, and CD8^+^ T cells in pan-cancer (Figure 4B). These results were further supported by published data and the CIBERSORT database (Figure 4C and Supplementary Figure S5E). It is widely acknowledged that the TME encompasses a diversity of components, including immune cells, stromal cells, and matrix. The immunosuppressive microenvironment is characterized by different immune cells such as Treg cells, MDSCs, and cancer-associated fibroblasts (CAFs), which contribute to a poorer response to immunotherapy. Therefore, we also explored the relationship between RBFOX2 and CD8^+^ T cells, Treg cells, CAFs, and MDSCs using various databases. The findings presented in Supplementary Figure S6A–S6D unveil that RBFOX2 showcased a negative correlation pertaining to the infiltration of CD8^+^ T cells, whereas it demonstrated a positive association with the infiltration of CAF and MDSC in the majority of cancer types. Actually, RBFOX2 shows positive correlation with Treg in majority of cancer types in QUANTISEQ, while shows negative correlation with Treg in some cancer types in CIBERSORT.

**FIGURE 5:**
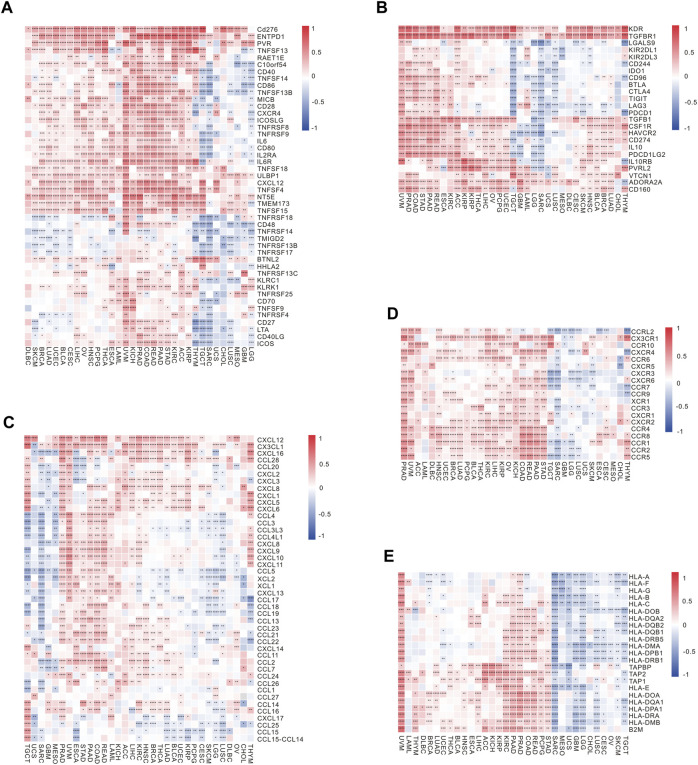
Correlation of RBFOX2 expression with immunoregulation-related genes. **(A)** The correlation heatmap between expression of RBFOX2 and immune activating genes. **(B)** The correlation heatmap between expression of RBFOX2 and immunosuppressive genes. **(C)** The correlation heatmap between expression of RBFOX2 and chemokine genes. **(D)** The correlation heatmap between expression of RBFOX2 and chemokine receptor genes. **(E)** The correlation heatmap between expression of RBFOX2 and MHC genes. MHC, histocompatibility complex; *, *p* < 0.05; **, *p* < 0.01; ***, *p* < 0.001, ****, *p* < 0.0001.

In tumor immunotherapy, the vital contribution of immune checkpoint-related genes cannot be overlooked. A gene co-expression analysis was carried out in this research to examine the connections between RBFOX2 and immune active genes as well as genes immunosuppressive genes. A significant correlation between RBFOX2 and immune active genes was shown in Figure 5A. Furthermore, RBFOX2 expression showed a significant association with immunosuppressive genes like PD-L1, CTLA4, VTCN1, KDR, and TGFBR1 across different types of cancers (Figure 5B). Notably, RBFOX2 expression exhibited a positive association with a wide range of chemokine-related genes and genes related to chemokine receptors in the majority of cancer cases. However, in TGCT, SARC, GBM, LUSC, and LGG, RBFOX2 expression was negatively associated with the expression of certain chemokine-related genes and chemokine receptor-related genes (Figures 5C,D). Additionally, RBFOX2 expression exhibited a positive association with the majority of MHC-associated genes in the majority of cancers, but a negative association was observed in TGCT, SKCM, OV, CESC, LUSC, CHOL, GBM, LGG, UCS, MESO, and SARC (Figure 5E). To summarize, our findings provided compelling evidence for a substantial relationship between RBFOX2 expression and diverse immune constituents, encompassing immune cells, immune checkpoint genes, chemokine and chemokine receptor pathways, along with MHC-associated genes. These results propose that RBFOX2 could potentially partake in the TME and serve as a potential indicator for distinct immunotherapy responses in disparate cancer classifications.

**FIGURE 6:**
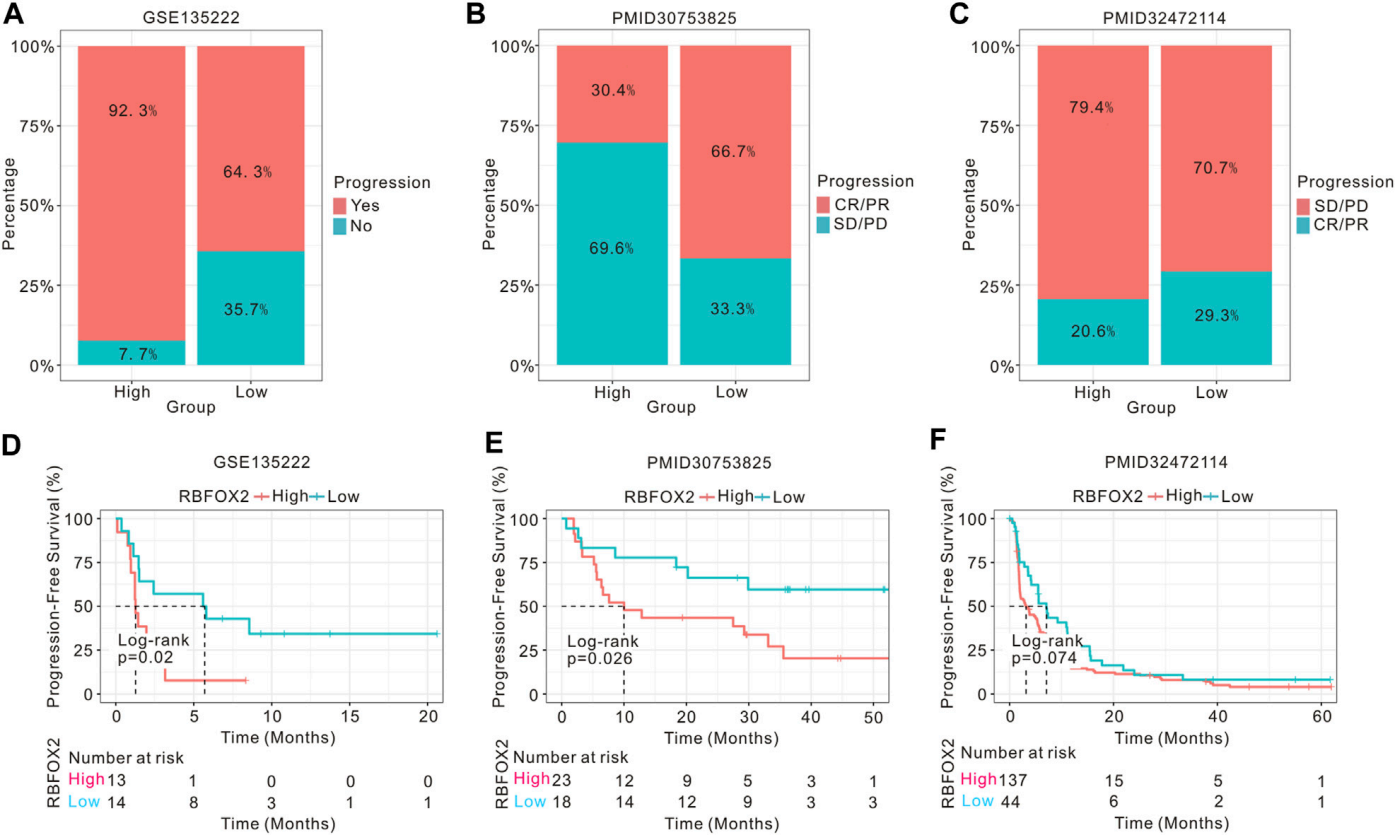
High expression of RBFOX2 is correlated to the prognosis of tumor patients undergoing immunotherapy. **(A)** Assessment of the immunotherapy response of NSCLC patients related to RBFOX2 expression using GSE135222 dataset. **(B)** Assessment of the immunotherapy response of melanoma patients related to RBFOX2 expression using public dataset (PMID30753825). **(C)** Assessment of the immunotherapy response of ccRCC patients related to RBFOX2 expression using public database (PMID32472114). **(D)** Assessment of the PFS of NSCLC patients with immunotherapy using GSE135222 dataset. **(E)** Assessment of the PFS of melanoma patients with immunotherapy using public dataset (PMID30753825). **(F)** Assessment of the PFS of ccRCC patients with immunotherapy using public database (PMID32472114). NSCLC, non-small cell lung cancer; ccRCC, clear cell renal cell carcinoma; PFS, progression-free survival; CR, complete response; PR, partial response; SD, stable disease; PD, progressive disease.

To investigate the association between RBFOX2 expression and the prognosis of tumor patients receiving immunotherapy, we conducted an analysis on the prognostic outcomes and immunotherapy response of patients with non-small cell lung cancer (NSCLC), ccRCC, and melanoma, categorized by their RBFOX2 expression levels. Our findings supported our hypothesis, we discovered a notable association between elevated expression levels of RBFOX2 and decreased response to treatment, along with unfavorable progression-free survival (PFS) outcomes in patients with advanced NSCLC and melanoma who received immunotherapy (Figures 6A,B,D,E). However, there was no difference in PFS among ccRCC patients who received immunotherapy, regardless of their RBFOX2 expression levels (Figures 6C,F).

### 3.4 Functional enrichment analysis of RBFOX2

Functional enrichment analysis was further performed to study the biological value and signaling pathways of RBFOX2 expression in various cancers. The gene-gene interaction network exhibited top 20 genes associated with RBFOX2 performed with Genemania and exhibited 10 interacted genes via STRING (Supplementary Figure S7A,B). Moreover, GO analysis revealed that top pathways influenced by RBFOX2 were regulation of RNA splicing, translesion synthesis, postreplication repair and DNA synthesis involved in DNA repair achieved by Genemania (Supplementary Figure S7C). The consistent results were performed by STRING, which indicated the RBFOX2 was associated with RNA splicing (Supplementary Figure S7D). Subsequently, KEGG pathway revealed that RBFOX2 expression was correlated with neurodegeneration-multiple diseases, mismatch repair, DNA replication, DNA replication, homologous recombination, fanconi anemia pathway, spinocerebellar ataxia, breast cancer and pathogenic *Escherichia coli* infection, as well as associated with spliceosome (Supplementary Figure S7E,F).

Considering the important role of metabolic reprogramming in tumor development, we analyzed the association of RBOFX2 with metabolic pathways in pan-cancer. As shown in Supplementary Figure S9, RBFOX2 expression was involved in energy metabolism, amino acid metabolism, lipid metabolism and nucleotide metabolism such as oxidative phosphorylation, rug metabolism, quinone biosynthesis, inositol phosphate metabolism, mannose type O-glycan biosynthesis. These results suggested that RBFOX2 may influence the development of pan-cancer by influencing the metabolic reprogramming.

### 3.5 DNA methylation of RBFOX2 in pan-cancer

**FIGURE 7:**
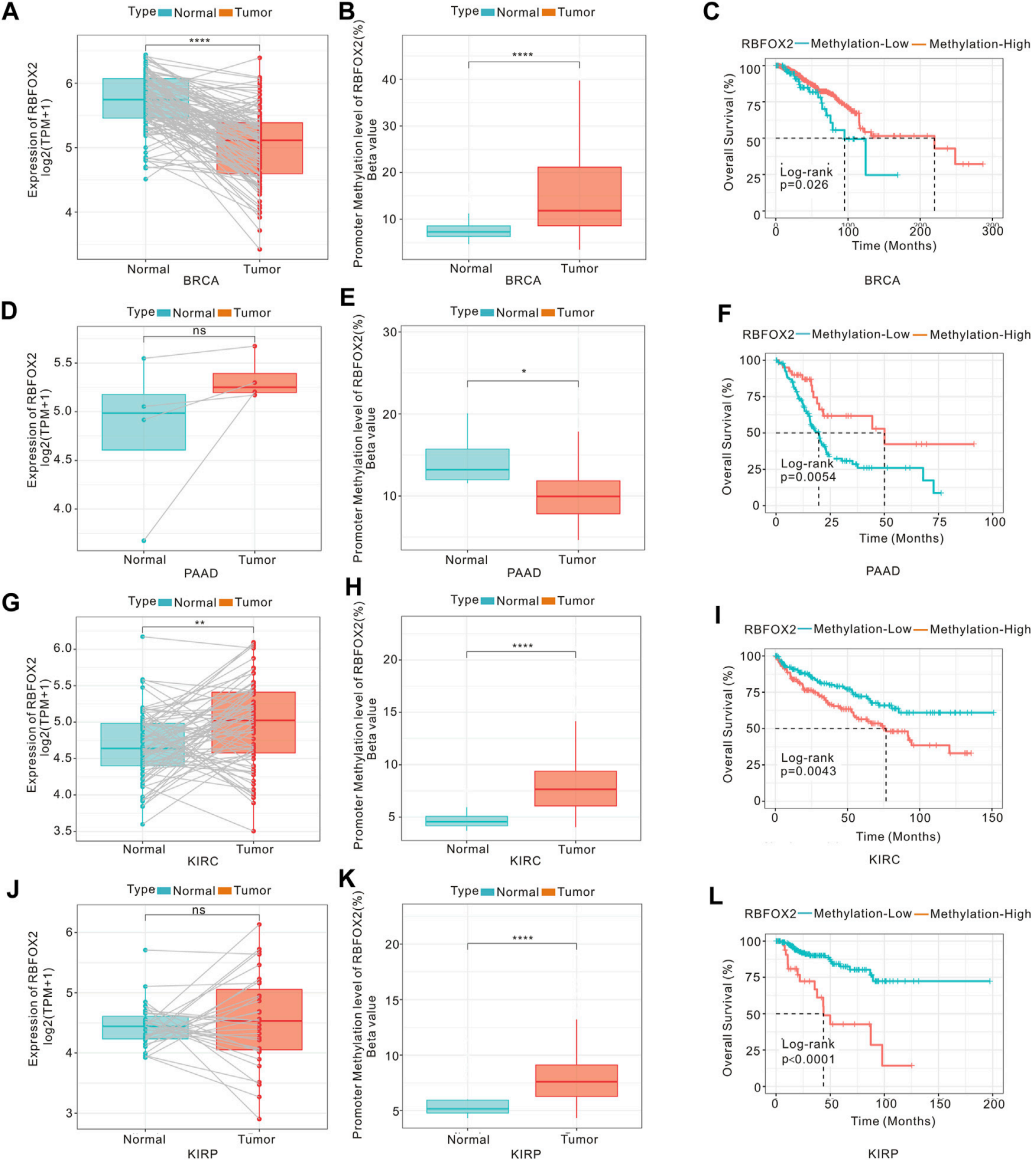
DNA methylation, RBFOX2 expression and OS of tumors. The mRNA expression **(A)** and mean DNA methylation at promoter regions **(B)** of RBFOX2 in BRCA. **(C)** The relationship between gene methylation of RBFOX2 and OS in BRCA patients. The expression **(D)** and mean DNA methylation at promoter regions **(E)** of RBFOX2 in PAAD. **(F)** The relationship between gene methylation of RBFOX2 and OS in PAAD patients. The expression **(G)** and mean DNA methylation at promoter regions **(H)** of RBFOX2 in KIRC. **(I)** The relationship between gene methylation of RBFOX2 and OS in KIRC patients. The expression **(J)** and mean DNA methylation at promoter regions **(K)** of RBFOX2 in KIRP. **(L)** The relationship between gene methylation of RBFOX2 and OS in KIRP patients. OS, overall survival; *, *p* < 0.05; **, *p* < 0.01; ****, *p* < 0.0001.

DNA methylation is an epigenetic alterations mechanism and could control the gene expression. Therefore, we conducted an analysis on the correlation between DNA methylation and the expression of RBFOX2 in various types of cancers. Results from Supplementary Figure S9 showed that the level of DNA methylation in RBFOX2 was higher in BRCA, CESC, HNSC, KIRC, KIRP, LUAD, PRAD, and UCEC, while it was lower in CHOL, PAAD, and THCA. Additionally, we conducted a K-M analysis to assess the relationship between DNA methylation and the prognosis of patients with pan-cancer. As shown in Supplmentary Figure S10, RBFOX2 methylation was a poor prognostic for KIRC, KIRP, LIHC, PCPG, TGCT, THCA, and UCS, but a protective factor for BLCA, BRCA, CESC, COAD, DLBC, HNSC, LAML, LGG, LUAD, PAAD, SKCM, and THYM. The RBFOX2 gene expression and promoter methylation in BRCA, PAAD, KIRC as well as KIRP were shown in Figure 7. We observed RBFOX2 gene expression was lower in BRCA tissues, while its promoter methylation was higher in BRCA tissues (Figures 7A,B). It is suggested that the decrease in the expression of RBFOX2 may be due to its promoter methylation. Additionally, a lower promoter methylation of RBFOX2 was found to be associated with worse OS for BRCA patients (Figure 7C). In the case of PAAD, RBFOX2 expression did not exhibit any noteworthy differences between tumor tissues and their matched normal tissues (Figure 7D). However, the promoter methylation was lower in tumor tissues, and a lower promoter methylation of RBFOX2 was associated with worse OS for BRCA patients (Figures 7E,F). On the other hand, in KIRC (Figures 7G,H), both RBFOX2 gene expression and promoter methylation were higher in tumor tissues, suggesting the involvement of other mechanisms in increasing the expression level of RBFOX2. Further experiments are needed to analyze these mechanisms. Moreover, a higher promoter methylation of RBFOX2 indicated worse OS for KIRC patients (Figure 7I). In the case of KIRP, there was no difference in RBFOX2 expression between tumor tissues with paired normal tissues (Figure 7J). However, the promoter methylation was higher in tumor tissues, and a higher promoter methylation of RBFOX2 indicated worse OS for KIRP patients (Figures 7K,L).

## 4 Discussion

Previous studies have investigated the expression and biological effect of RBFOX2 in some tumors, such as nasopharyngeal carcinoma (Luo et al., 2021), endometrial cancer (Zhang et al., 2022) and ovarian cancer (Gordon et al., 2019). Nevertheless, the untapped prognostic capacity and immunological significance of RBFOX2 in the scope of pan-cancer remains untapped. In this particular investigation, a thorough examination of RBFOX2 in 33 diverse cancer classifications was conducted. The findings revealed disparate expressions of RBFOX2 and its correlation with adverse prognosis in the majority of malignancies. Furthermore, RBFOX2 showed a close correlation with immune infiltration cells, immune checkpoints, chemokines, and chemokine receptors, suggesting its association with the TME and indicating a poor response to immunotherapy in cancers. Moreover, DNA methylation was found to be correlated with RBFOX2 expression in most tumors, and RBFOX2 methylation was identified as a protective factor for BLCA, BRCA, CESC, COAD, DLBC, HNSC, LAML, LGG, LUAD, PAAD, SKCM, and THYM. Furthermore, we constructed the GO and KEGG analysis and found that RBFOX2 was closely associated with RNA splicing.

RBFOX2, a well-characterized regulator of alternative splicing, has been reported involved in EMT and contributes to metastasis of several cancers (Yeo et al., 2009; Braeutigam et al., 2014). RBFOX2 was overexpression and promoted the migration of gastric cancer cells (Ou et al., 2021), which was consistent with what we have found that RBFOX2 was high in STAD tissues than normal tissues. The mRNA level of RBFOX2 was significantly upregulated in nasopharyngeal carcinoma (NPC) tissues, correlating with the initiation of NPC cell tumorigenesis (Luo et al., 2021). Suppression of RBFOX2 significantly decreased the growth and survival of lymphoid malignancies by regulating aberrant alternative splicing of ARNT (Cooper et al., 2022), which was consistent in this study that RBFOX2 was high in DLBC tissues. Knockdown of RBFOX2 significantly upregulation of the KIF1B and increased sensitivity to anoikis in ovarian cancer cells (Gordon et al., 2019). Silencing RBFOX2 also significantly inhibited cell proliferation and invasion in laryngeal cancer by mediating MENA alternative splicing (Lu et al., 2023). Furthermore, RBFOX2 expression was found to increase during the acquisition of a mesenchymal phenotype in breast cancer cells (Shapiro et al., 2011). Despite previous study and our results found that the downregulation of RBFOX2 mRNA in colorectal cancer tissue compared to normal tissue in the colon cancer TCGA data (Danan-Gotthold et al., 2015), RBFOX2 protein expression was found to be upregulated in colorectal cancer (Choi et al., 2019). Recently, Jbara *et al.* reported that RBFOX2 acted as a metastatic suppressor in pancreatic cancer and discovered a signature of RBFOX2-regulated alternative splicing in metastatic pancreatic cancer (Jbara et al., 2023). In addition, previous study has showed a significant increase RBFOX2 expression in pancreatic cancers compared to normal pancreas (Maurin et al., 2023), which was consistent with our results. Taken together, these findings suggested that RBFOX2 may exhibit either oncogenic or suppressive properties depending on the specific tumor type. We observed differential expression of RBFOX2 in pan-cancer, indicating its significant and crucial role in tumor development.

This research aimed to explore the correlation between RBFOX2 expression and OS, DSS, DFI, and PFI in a wide range of cancers. Our K-M analysis revealed that RBFOX2 is a risk factor for OS of patients with ACC, BLCA, CESC, HNSC, KICH, LAMI, LUAD, MESO, OV, PAAD, SARC, STAD, THCA, THYM, UCEC, and UVM, while it acts as a protective factor for OS of patients with CHOL, ESCA, KIRC, LGG, PCPG, READ, SKCM, and UCS. Notably, previous research has also shown that RBFOX2 linked with poor prognosis of NPC patients (Luo et al., 2021), which aligns with our findings of RBFOX2 indicating poor OS for HNSC patients. Furthermore, our study is consistent with another study that found high levels of RBFOX2 was associated with worse survival in gastric cancer patients (Ou et al., 2021). These collective results suggested that higher RBFOX2 expression equivalent to a protective role in most tumor types.

Recently, immune checkpoint blockade (ICB) therapy, which targets the dysfunctional immune system and induces cancer-cell killing by CD8-positive T cells, has been reported not only to revolutionize the field of cancer treatment but has also establish itself as a crucial immunotherapy option for combating cancer (Abril-Rodriguez and Ribas, 2017). However, the efficacy of ICBs is limited due to genomic alterations, suppressed antigen presentation, and higher heterogeneity of the TME (Pitt et al., 2016; Vitale et al., 2021; Chhabra and Weeraratna, 2023). The TME comprises a diverse array of components, including immune cells, stromal cells, extracellular matrix, cytokines, and various other factors. The conditions created within the TME play a pivotal role in the initiation, progression, invasion, and metastasis of tumors. Furthermore, these conditions are intricately connected to the survival of tumor cells. Immune cells within the TME have been reported can either promote or suppress tumor growth and are linked to the advancement of cancer cells. Nevertheless, limited attention has been given to exploring the connection between RBFOX2 and the TME. Only one study demonstrated that RBFOX2 was negatively associated with CD8^+^ T cells and M2 macrophages, and was also associated with worse ICB response (Bareche et al., 2022). Additionally, it has been reported that 20% of TME-associated alternative splicing is regulated by QKI and RBFOX2 (Brosseau et al., 2014). In this particular investigation, the correlation between RBFOX2 and the TME was thoroughly explored. The results demonstrated a significant association between RBFOX2 expression and essential aspects of the TME, such as immune score, stromal score, and tumor purity across multiple cancer types. This suggests that RBFOX2 may participate the development by interacting with the TME. Additionally, the study analyzed the correlation between RBFOX2 and immune cells such as CD8^+^ T cells, Tregs, tumor-associated macrophages, and NK cells, which are known as immunotherapy related cells (Lin et al., 2020; Xu et al., 2021; Song et al., 2022; Larrayoz et al., 2023). The findings showed that RBFOX2 was linked to CD8^+^ T cells, CAFs, and Tregs in different types of cancer. Furthermore, RBFOX2 was found to be significantly associated with immune activating genes, immunosuppressive genes, chemokines, chemokine receptors, and MHC. These notable findings strongly suggest that RBFOX2 expression holds pronounced significance in the context of the TME, potentially influencing patient prognosis. Additionally, the identification of RBFOX2 as a novel target paves the way for the development of innovative immunosuppressive therapies.

Furthermore, to sustain uncontrolled growth and proliferation, the cancer cells keep metabolism reprogramming. Cancer cells could change the oxidative phosphorylation to aerobic glycolysis, Warburg Effect and increased lipid uptake, lipogenesis as well as cholesterol synthesis (Schiliro and Firestein, 2021). Metabolic reprogramming supports high energy production for cancer cells in migration and metastasis (Zanotelli et al., 2021). In addition, metabolic reprogramming of cancer and immune cells determines the cancer anti-tumor immune response (Xia et al., 2021). Wu *et al.* developed scMetabolism for biologists to easily quantify metabolic activity by using scRNA-seq data (Wu et al., 2022), as well as illustrated the metabolic activity of MRC1^+^CCL18^+^ macrophages was increased in the colorectal cancer liver metastasis. We analyzed the association between RBFOX2 and metabolic reprogramming and found RBFOX2 involved in most of metabolic pathways in pan-cacer. Future study needs scMetabolism database to explore the metabolic reprogramming in single cell level.

DNA methylation is an important form of epigenetic alteration that could regulate gene expression by affecting gene stability (Moore et al., 2013). This can be achieved through various mechanisms, such as altering the DNA conformation and chromatin structure (Wang et al., 2021). In particular, DNA methylation is often associated with the inhibition of tumor suppressor genes in malignant conditions (Saghafinia et al., 2018; Mehdi and Rabbani, 2021). The investigation made use of both the DNMIVD and CPTAC datasets to examine the methylation status of RBFOX2 in diverse cancer types. Our findings indicate that RBFOX2 methylation is closely linked to OS, DSS and PFS in certain tumor patients. In fact, RBFOX2 methylation appears to have a protective effect in cancers such as BLCA, BRCA, CESC, COAD, DLBC, HNSC, LAML, LGG, LUAD, PAAD, SKCM, and THYM. However, further research is needed to validate the potential role of RBFOX2 methylation in these cancers.

The results presented in the previous section highlight the significant prognostic and immune value of RBFOX2 in pan-cancer. HCC is particularly relevant in this context, as it represents a substantial patient population and is the fourth leading cause of cancer-related death in China (Yang et al., 2019). Furthermore, previous studies have identified RBFOX2 as a key splicing factor in hepatitis C virus (HCV) related liver cancer (Cai et al., 2020). Therefore, HCC can be considered a representative cancer for further investigation. In this study, IHC experiments revealed that RBFOX2 expression was lower in tumor tissues compared to paired adjacent tissues. However, our analysis of TCGA, GTEx, and CPTAC databases demonstrated that RBFOX2 expression was higher in LIHC tumor tissues compared to normal tissues. It is reason that the differences in results may be attributed to the fact that the IHC experiment utilized HCC patient tumor tissues and paired adjacent tissues, whereas the TCGA, GTEx, and CPTAC databases used non-paired tissues. Moreover, our study firstly revealed that knockdown of RBFOX2 could promote the proliferation and metastasis of HCC cells *in vitro*, indicating that RBFOX2 functions as a suppressor gene for liver cancer. Further studies are required to elucidate the underlying molecular mechanisms.

To conclude, we conducted an investigation on the expression of RBFOX2 in different types of cancer and its correlation with patient prognosis. Our findings indicate that RBFOX2 has the potential to serve as a prognostic marker for various tumors. Furthermore, we specifically focused on exploring the interplay among DNA methylation, RBFOX2 expression and prognosis in pan-cancer. Our results suggest that variations in promoter methylation levels might contribute to the differential expression of RBFOX2 in pan-cancer. Specifically, we observed lower RBFOX2 levels in tumor tissues of HCC patients in comparison to neighboring tissues. *In vitro* experiments provided evidence that knocking down RBFOX2 significantly increased the growth and metastasis of liver cancer cells. Moreover, we discovered a strong link between RBFOX2 and immune infiltration cells, immune checkpoints, chemokines, and chemokine receptors within the TME. Noteworthy is the fact that differential RBFOX2 expression affected the response to immunotherapy and patient prognosis. To summarize, our findings strongly suggest that RBFOX2 plays a vital role in the TME and has the potential to serve as a promising therapeutic target for enhancing the efficacy of immunotherapy.

## Data Availability

The datasets presented in this study can be found in online repositories. The names of the repository/repositories and accession number(s) can be found in the article/Supplementary Material.
