# Supplementary material for: Integrated pan-cancer analysis reveals the immunological and prognostic potential of RBFOX2 in human tumors
Source: Front Pharmacol. 2024 May 31;15:1302134. doi: 10.3389/fphar.2024.1302134 (PMC11176534; doi:10.3389/fphar.2024.1302134)
Supplement: Supplementary file 1 [file DataSheet1.docx]

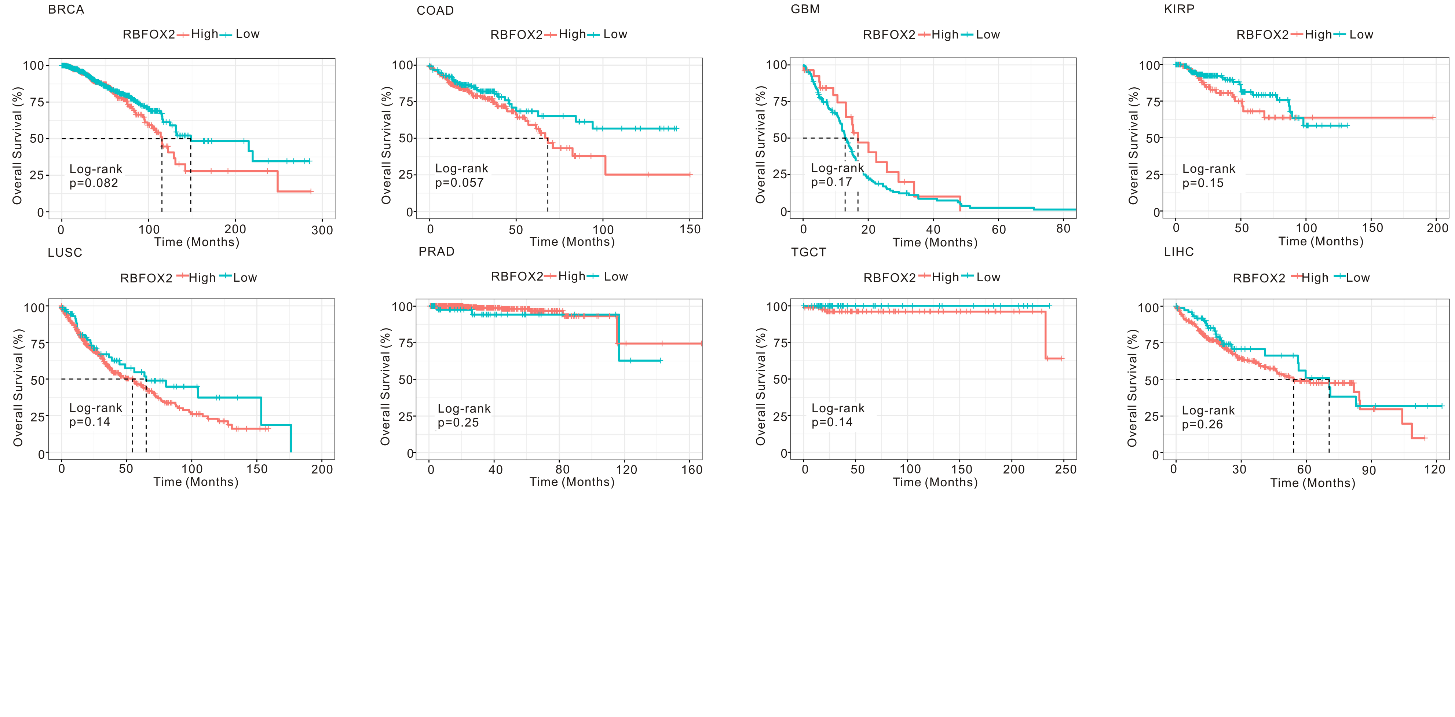


**Supplementary Figure 1. Association of RBFOX2 expression with OS in pan-cancer.** K-M examination of the relationship between the OS and RBFOX2 expression from TCGA database. The optimal cut-off value of expression of RBFOX2 was determined by surv_cutpoint function. K-M, Kaplan-Meier; OS, overall survival.


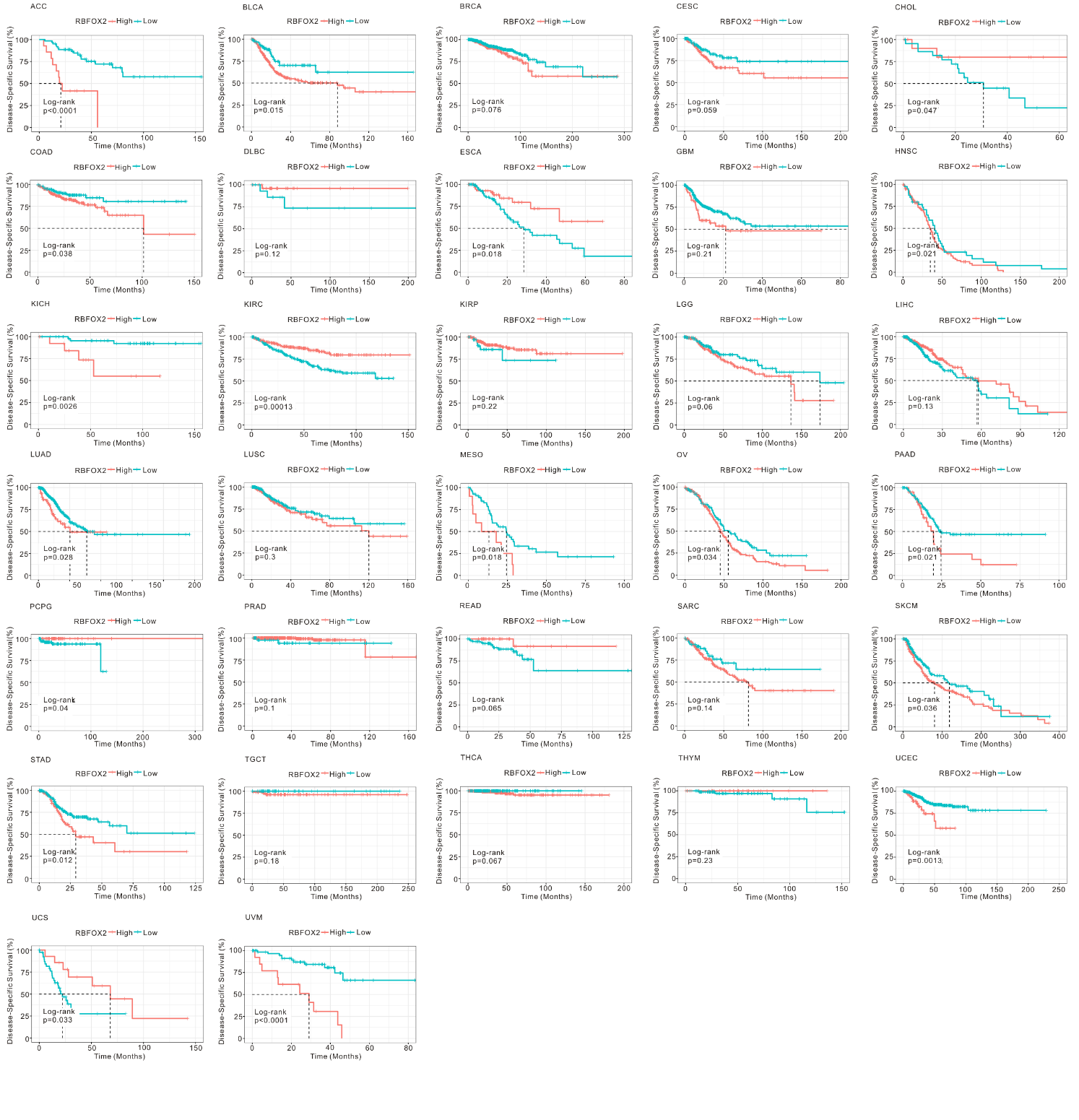


**Supplementary Figure 2. Association of RBFOX2 expression with DSS in pan-cancer.** K-M examination of the relationship between the DSS and RBFOX2 expression from TCGA database. The optimal cut-off value of expression of RBFOX2 was determined by surv_cutpoint function. K-M, Kaplan-Meier; DSS, disease specific survival.

**
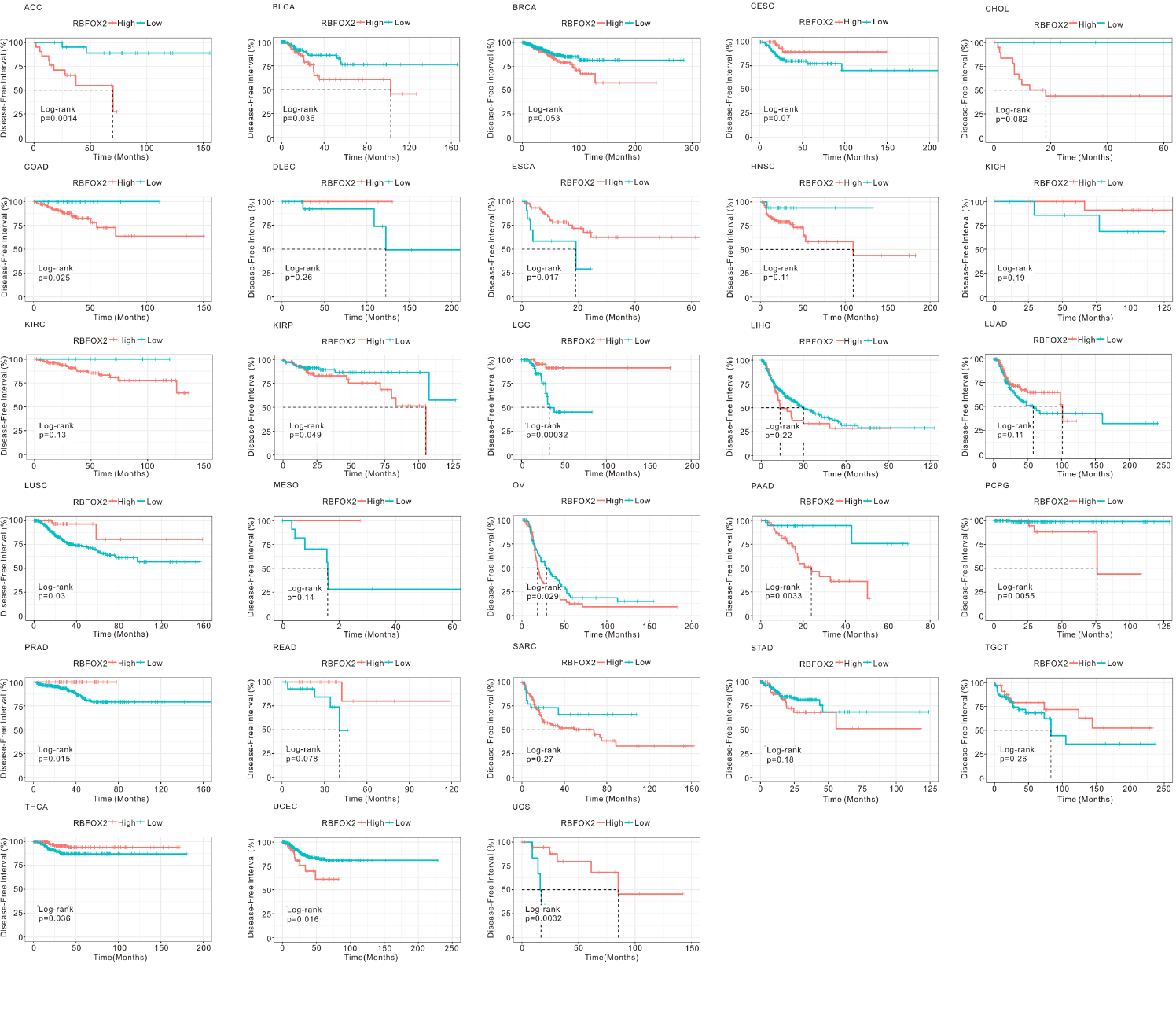
**

**Supplementary Figure 3 Association of RBFOX2 expression with DFI in pan-cancer.** K-M examination of the relationship between the DFI and RBFOX2 expression from TCGA database. The optimal cut-off value of expression of RBFOX2 was determined by surv_cutpoint function. K-M, Kaplan-Meier; DFI, disease free interval.


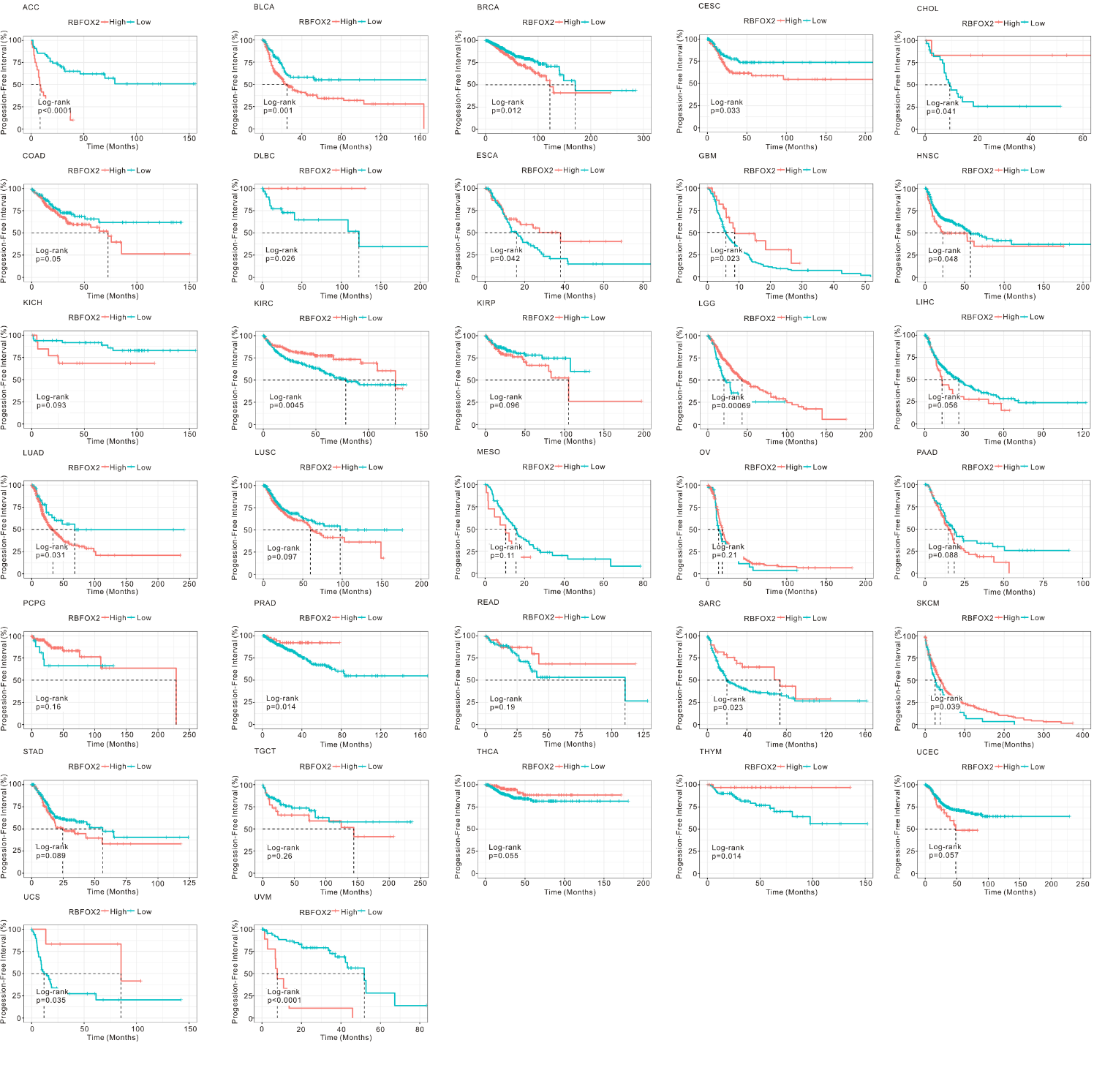


**Supplementary Figure 4. Association of RBFOX2 expression with PFI in pan-cancer.** K-M examination of the relationship between the PFI and RBFOX2 expression from TCGA database. The optimal cut-off value of expression of RBFOX2 was determined by surv_cutpoint function. K-M, Kaplan-Meier; PFI, progression free interval.


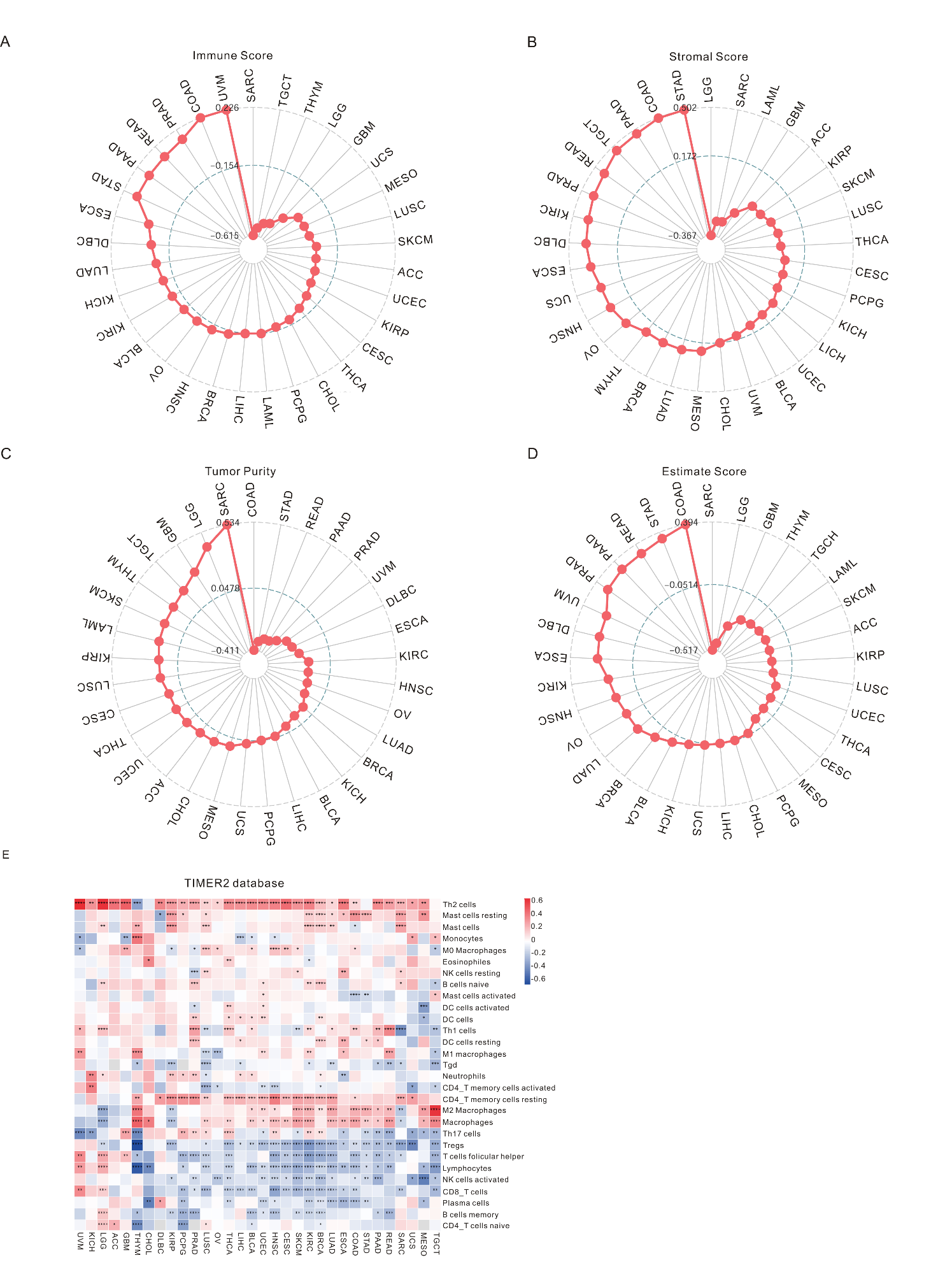


**Supplementary Figue 5. Relationship of RBFOX2 expression with tumor microenvironment and immune cell infiltration.** (A) Examining the association between RBFOX2 expression and tumor Immune Score in pan-cancer. (B) Examining the association between RBFOX2 expression and tumor Stromal Score in pan-cancer. (C) Examining the association between RBFOX2 expression and Tumor Purity in pan-cancer. (D) Examining the association between RBFOX2 expression and Estimate Score in pan-cancer. (E) Examining the association between RBFOX2 expression with immune cell infiltration using the TIMER2 database. *, *P* < 0.05; **, *P* < 0.01; ***, *P* < 0.001, ****, *P* <0.0001.


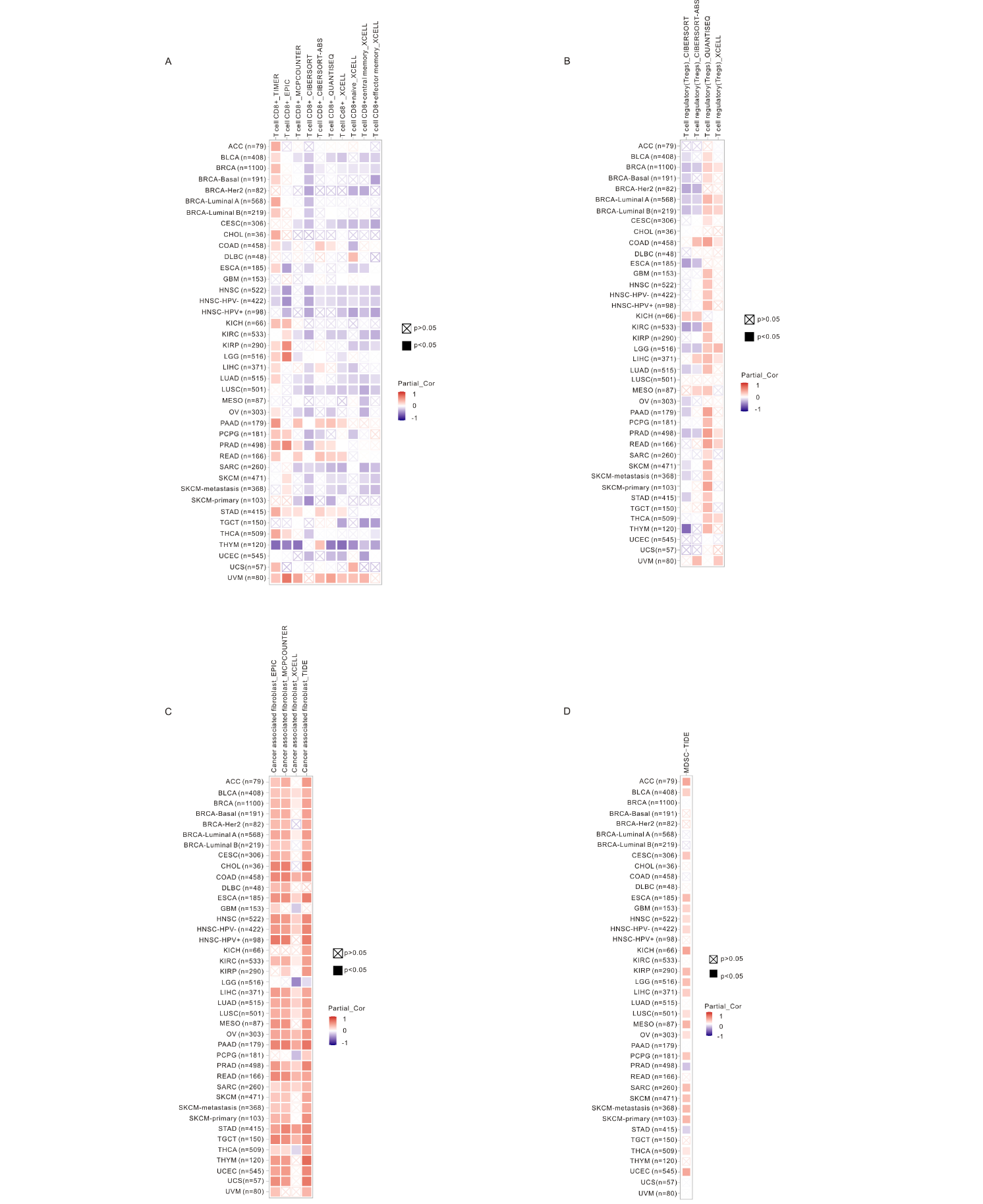


**Supplementary Figure 6. Correlation of RBFOX2 expression with infiltration level of immune cells.** (A) Association of RBFOX2 expression and infiltration level of CD8+ T cells. (B) Association of RBFOX2 expression and infiltration level of T cell regulatory (Tregs). (C) Association of RBFOX2 expression and infiltration level of cancer associated fibroblast (CAF) cells. (D) Association of RBFOX2 expression and infiltration level of myeloid-derived suppressor cell (MDSC) cells.

**
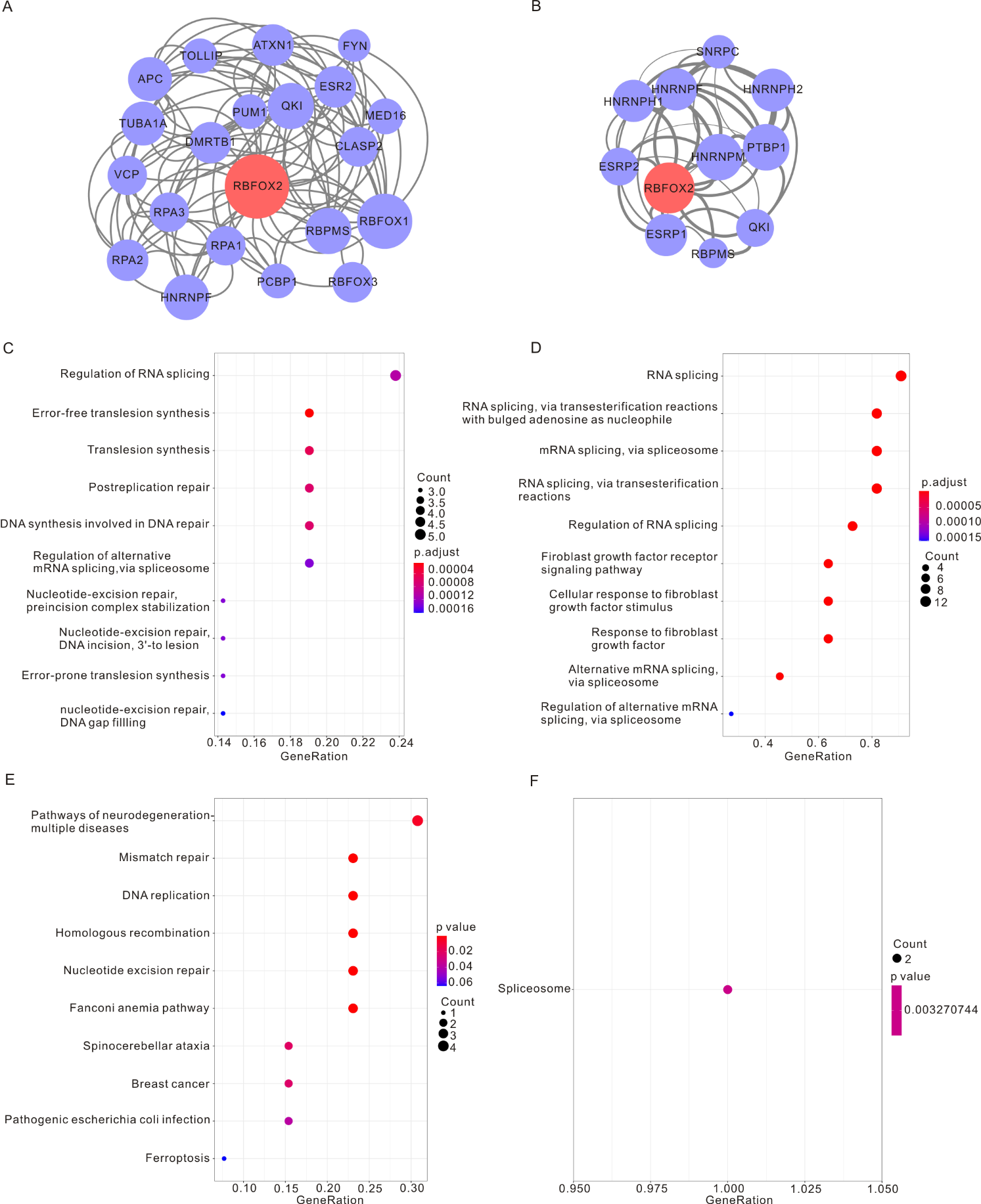
**

**Supplementary Figure 7. Network and enrichment analyses.** (A) Construction of PPI network involved in 20 RBFOX2-interacting proteins based on Genemina. (B) Construction of gene-gene network based on STRING. Each edge is present as a different line width according to its degree score representing the extent of interaction between each two nodes. (C) The functional enrichment analysis based on KEGG pathways using PPI interaction network from Genemina. (D) The functional enrichment analysis based on KEGG pathways using PPI interaction network from STRING. (E) The GO analysis of RBFOX2- interacting proteins based on Genemina. (F) The GO analysis of RBFOX2- interacting proteins based on STRING. PPI, protein-protein interactions; GO, gene ontology; KEGG, kyoto encyclopedia of genes and genomes.

**
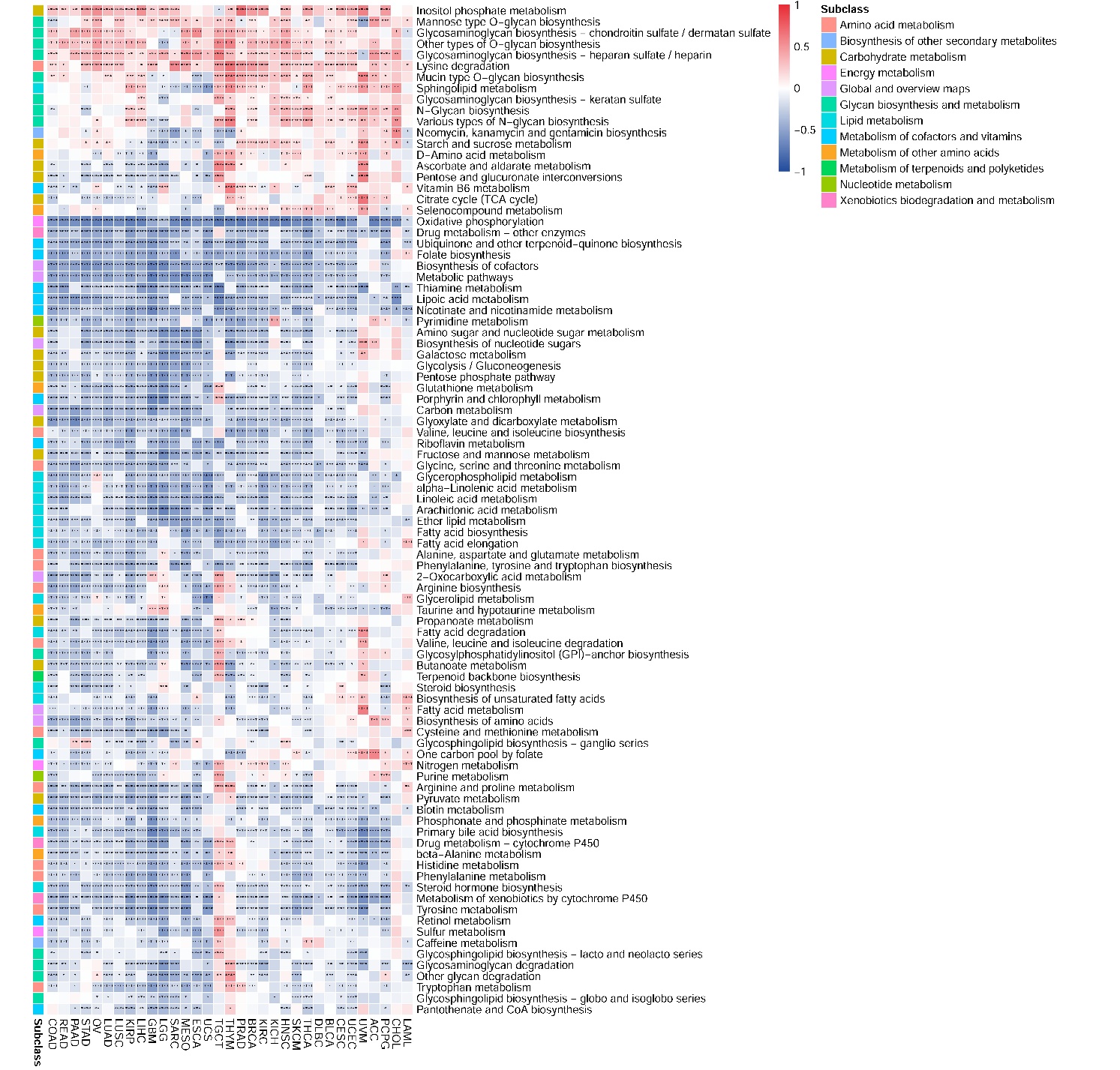
**

**Supplementary Figure 8. Association of RBOFX2 expression with metabolic pathways in pan-cancer.** *, *P* < 0.05; **, *P* < 0.01; ***, *P* < 0.001, ****, *P* <0.0001.

**
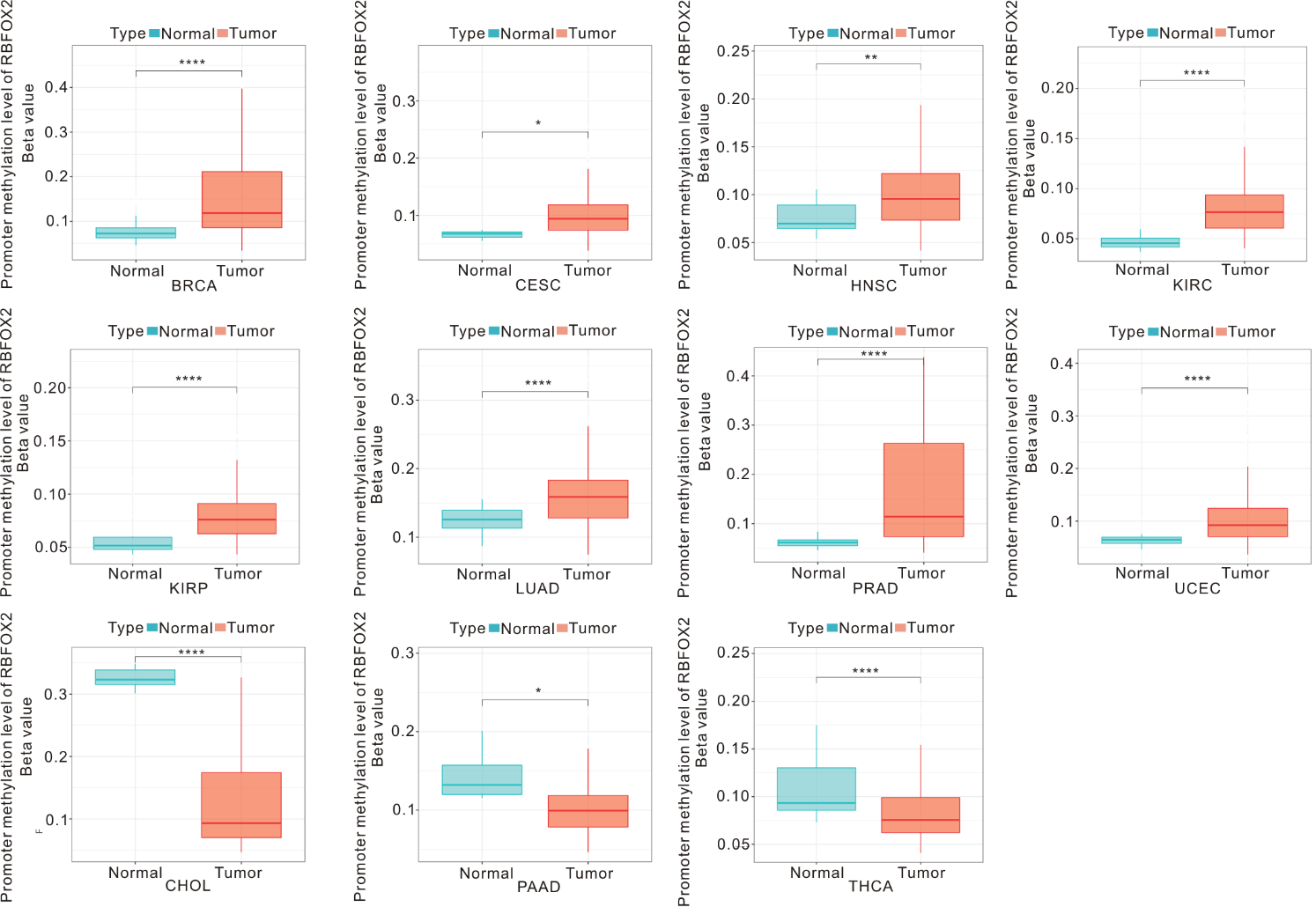
**

**Supplementary Figure 9. DNA methylation patterns of RBFOX2.** *, P < 0.05; **, P < 0.01; ****, P <0.0001.


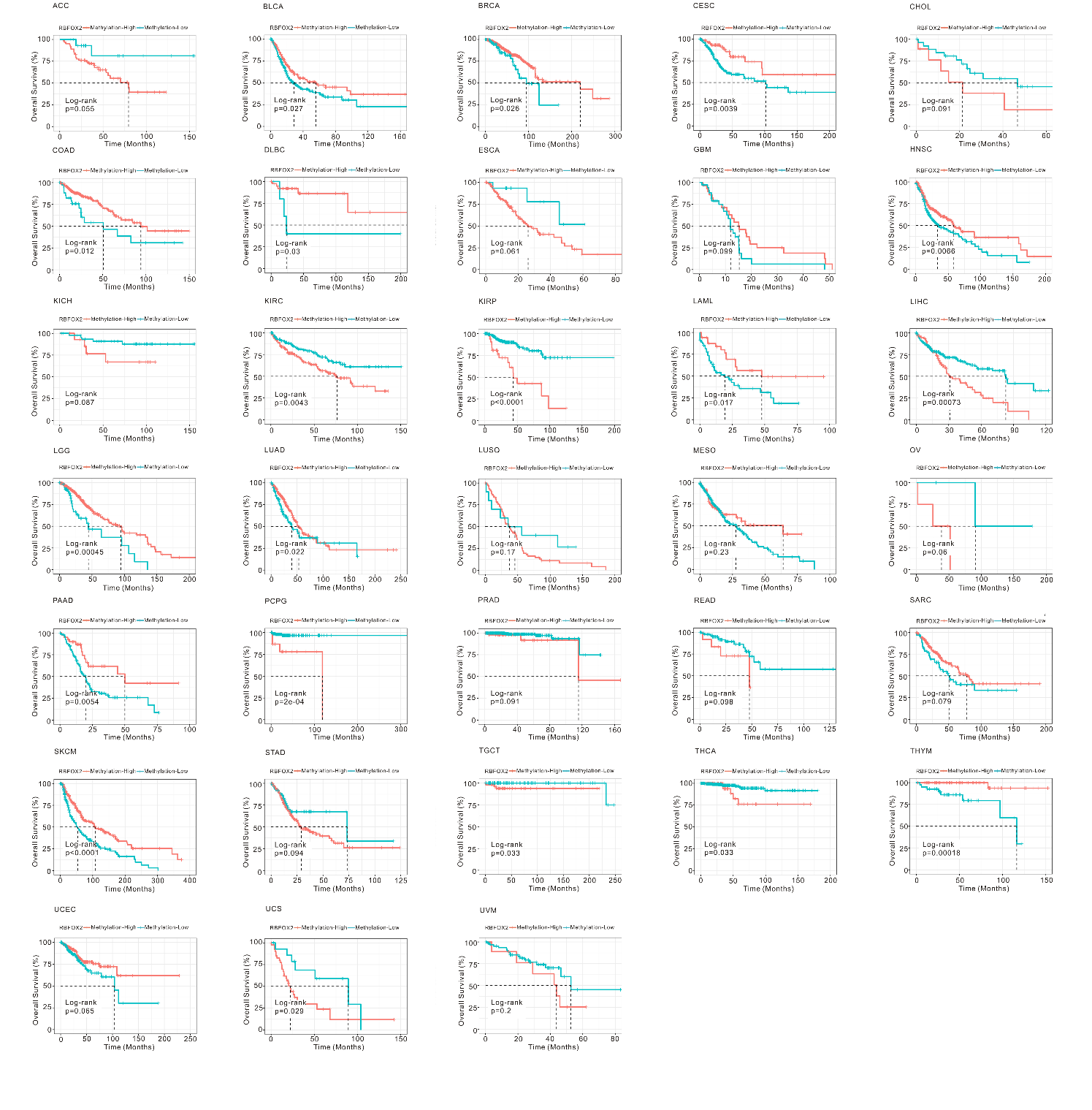


**Supplementary Figure 10. Correlation between RBFOX2 methylation and OS of various tumors.** The optimal cut-off value of mean DNA methylation at promoter regions of RBFOX2 was determined by surv_cutpoint function. OS, overall survival.

**Supplementary Table 1** Association of RBFOX2 expression and prognostic relationship in pan-cancer

| Tumor type | RBFOX2 mRNA expression | OS | PFI |
| --- | --- | --- | --- |
| ACC | Low | Negative | Negative |
| BLCA | Low | Negative | Negative |
| BRCA | Low | ns | Negative |
| CESC | Low | Negative | Negative |
| CHOL | High | Positive | Positive |
| COAD | Low | ns | ns |
| DLBC | High | Positive | Positive |
| ESCA | Low | Positive | Positive |
| GBM | Low | ns | Positive |
| HNSC | High | Negative | Negative |
| KICH | ns | Negative | ns |
| KIRC | High | Positive | Positive |
| KIRP | High | ns | ns |
| LAML | Low | Negative | ns |
| LGG | High | Positive | Positive |
| LIHC | High | ns | ns |
| LUAD | Low | Negative | Negative |
| LUSC | High | ns | ns |
| MESO | - | Negative | ns |
| OV | Low | Negative | ns |
| PAAD | High | Negative | ns |
| PCPG | High | Positive | ns |
| PRAD | Low | ns | Positive |
| READ | Low | Positive | ns |
| SARC | ns | Negative | Positive |
| SKCM | ns | Positive | Positive |
| STAD | High | Negative | ns |
| TGCT | Low | ns | ns |
| THCA | Low | Negative | ns |
| THYM | High | Negative | Positive |
| UCEC | Low | Negative | ns |
| UCS | Low | Positive | Positive |
| UVM | - | Negative | Negative |

ns, no signifance, -, no detection, OS, overall survival, PFI, progression free interval.
